# MOFs-based nanoagent enables dual mitochondrial damage in synergistic antitumor therapy via oxidative stress and calcium overload

**DOI:** 10.1038/s41467-021-26655-4

**Published:** 2021-11-04

**Authors:** Weier Bao, Ming Liu, Jiaqi Meng, Siyuan Liu, Shuang Wang, Rongrong Jia, Yugang Wang, Guanghui Ma, Wei Wei, Zhiyuan Tian

**Affiliations:** 1 School of Chemical Sciences, University of Chinese Academy of Sciences, 100049 Beijing, P. R. China; 2 State Key Laboratory of Biochemical Engineering, Institute of Process Engineering, Chinese Academy of Sciences, 100190 Beijing, P. R. China; 3 Department of Gastroenterology, Shanghai Tongren Hospital, Shanghai Jiao Tong University School of Medicine, 200336 Shanghai, P. R. China; 4 School of Chemical Engineering, University of Chinese Academy of Sciences, 100049 Beijing, P. R. China

**Keywords:** Drug development, Organic-inorganic nanostructures, Drug delivery

## Abstract

Targeting subcellular organelle with multilevel damage has shown great promise for antitumor therapy. Here, we report a core-shell type of nanoagent with iron (III) carboxylate metal-organic frameworks (MOFs) as shell while upconversion nanoparticles (UCNPs) as core, which enables near-infrared (NIR) light-triggered synergistically reinforced oxidative stress and calcium overload to mitochondria. The folate decoration on MOFs shells enables efficient cellular uptake of nanoagents. Based on the upconversion ability of UCNPs, NIR light mediates Fe^3+^-to-Fe^2+^ reduction and simultaneously activates the photoacid generator (pHP) encapsulated in MOFs cavities, which enables release of free Fe^2+^ and acidification of intracellular microenvironment, respectively. The overexpressed H_2_O_2_ in mitochondria, highly reactive Fe^2+^ and acidic milieu synergistically reinforce Fenton reactions for producing lethal hydroxyl radicals (•OH) while plasma photoacidification inducing calcium influx, leading to mitochondria calcium overload. The dual-mitochondria-damage-based therapeutic potency of the nanoagent has been unequivocally confirmed in cell- and patient-derived tumor xenograft models in vivo.

## Abstract (web summary)

Targeting damage to mitochondria has become an effective strategy antitumor therapies. Here, the authors report on nanoagents with upconversion nanoparticles as cores and photoacid-loaded MOFs as shells for NIR triggered Fenton reaction, acidification and calcium overload to provide synergistic mitochondrial damage.

## Introduction

The traditional single-modality antitumor strategy, such as chemotherapy and radiotherapy, generally encounters challenges such as adverse side effects and limited therapeutic efficacy^1–4^. These challenges highlight the vital significance of developing modalities capable of spatiotemporally controllably imparting multilevel damage to specific targets essential for tumor cell survival and proliferation^5^. For multiple-approach-involved therapeutic strategy, simultaneously triggering multiple actions is believed superior to the way with the involvement of individually stimulating because the former generally circumvents the complexity of treatment and brings about the spatial-temporal consistency of actions^6,7^. Among a wide range of triggering models, light, especially near-infrared (NIR) light with deeper-penetrating ability, has proven to be an appealing tool for precise activation of therapeutic activities with spatiotemporal controllability while without the need for physical contact^8,9^.

Besides the feature in terms of light-assisted simultaneously triggering multilevel antitumor mechanisms, the ability to guide antitumor action to vital subcellular organelles that are essential for cell survival and proliferation is unequivocally a key enabling factor for maximizing the overall therapeutic potency. Additionally, antitumor strategies undergoing tumor-targeted drug delivery and/or taking effects in response to tumor-associated biological cues have been proven efficient for spatially confining the range of action to target and therefore alleviating the damage to normal tissues^10,11^. Many evidences have revealed that mitochondria play key multifunctional roles in oncogenesis, including adenosine triphosphate (ATP) generation, redox and calcium homeostasis, and metabolic signal transduction, suggesting mitochondrion might be an ideal point of attack for antitumor action^12–14^. It is also noteworthy that mitochondria in tumor cells are generally characterized with distinctive properties, including hypoxia and upregulated hydrogen peroxide (H_2_O_2_)^15,16^. Thus, an opportunity has risen to develop new antitumor agents by targeting mitochondria and utilizing their site-specific species, the upregulated H_2_O_2_, for instance, as the endogenous stimulator for triggering the antitumor action, which is expected to confine the range of action locally to mitochondria and therefore present antitumor capabilities with the desired specificity and efficacy.

For site-specific attack of mitochondria, the chemodynamic therapy (CDT) based on oxidative stress of •OH species is characterized with unequivocal superiority owing to its underlying action mechanism^17^. Specifically, the Fenton reaction or Fenton-like reaction using H_2_O_2_ as the crucial substrate can be facilitated in the mitochondrial microenvironment due to the upregulated level of H_2_O_2_ species therein^15,16^. Moreover, as the crucial enabling species in CDT, •OH is characterized by much stronger oxidative stress and therefore potency for inducing mitochondrial damage than its counterpart active species involved in photodynamic therapy (PDT), namely, ^1^O_2_^18,19^. Additionally, the underlying mechanism of CDT using H_2_O_2_ as the substrate circumvents the intrinsic impediments originating from the tumor hypoxia that PDT typically encounters^20^.

Although CDT holds great promise for antitumor treatment based on the site-specific mitochondrial damage, several major impediments need to be overcome for enabling it practicality. Firstly, the Fenton reaction prefers an acidic environment, with an optimal reaction pH ranging from 2.0 to 5.0^21^, while the intracellular pH of tumor predominantly exhibits ~ 7.4^22^, which is not acidic enough to enable an efficient Fenton reaction and the accumulation of sufficient •OH for efficient CDT. Secondly, Fe^2+^ is typically more productive in generating •OH via the Fenton reaction but more unstable than Fe^3+ ^^23,24^, which makes efficient delivery of the productive Fe^2+^ into the tumor cytoplasm and the on-demand release of sufficient Fe^2+^ in situ remain a challenge. Additionally, for CDT-involved combined antitumor therapeutics, the simultaneous administration of Fenton reaction-including multiple approaches with different underlying pathways for mitochondrial damage is also challenging.

**Fig. 1:**
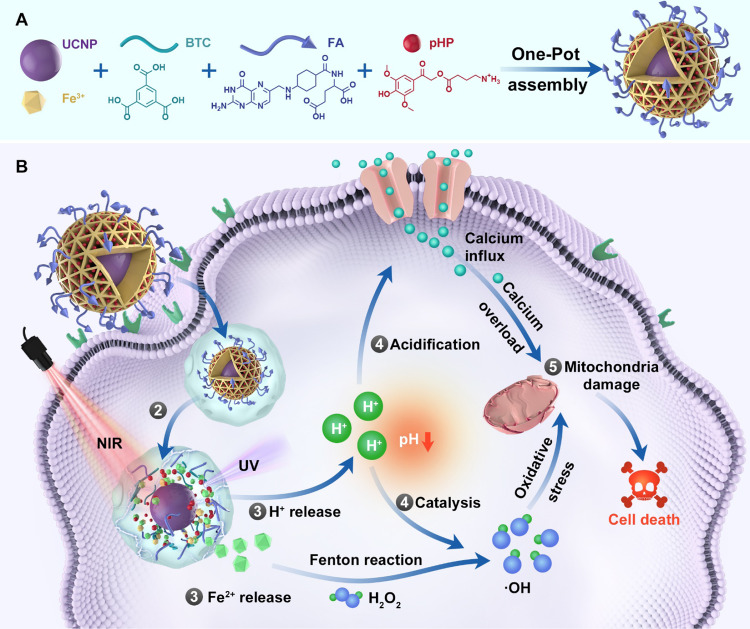
Schematic Illustration of the construction of FMUP nanoagent and the underlying anticancer mechanism. **A** Core–shell type FMUP nanoagents with UCNP as the core and photoacid (pHP) encapsulated in the cavity of FA-doped MOFs shell were constructed by one-pot self-assembly. The FMUP nanoagent was synthesized by coordination of carboxyl groups on BTC and FA with Fe^3+^. The UCNP as the core was located in the shell and simultaneously the pHP was loaded in the pore of the nanoagent. **B** Corresponding anticancer mechanism of FMUP nanoagent. ① Active internalization of FMUP in tumor cells helped by FA functionalization. ② Lysosome escape of FMUP induced by the increase of osmotic pressure after NIR light irradiation. ③ Fe^2+^ and H^+^ release from FMUP upon NIR light irradiation. ④ Photoacidification of intracellular microenvironment induced calcium influx and therefore calcium overload in the mitochondria and simultaneously generated a key acidic environment for efficient Fenton reactions. The release of Fe^2+^ and photoacidification synergistically reinforced Fenton reactions and therefore produced a large number of •OH within the close proximity of mitochondria. ⑤ As a result, the calcium overloaded and plentiful •OH enabled dual damage to mitochondria and further induced cell death.

Herein, we report a new type of MOFs-based core–shell nanoagent with NIR light-triggered potent antitumor capability based on the combination of Fenton reaction-enabled oxidative stress and an imbalance in calcium homeostasis (Fig. 1). In addition to its superiority regarding NIR light-triggering manner for circumventing the impediment of limited penetration depth of triggering light that ultraviolet (UV) or visible light typically suffers, this type of MOFs-based nanoagent is characterized with salient features in terms of the underlying antitumor mechanism. Specifically, folic acid (FA)-decoration and the site-specific species (H_2_O_2_) jointly enable confining the range of CDT locally to mitochondria of tumor cells. Additionally, triple enabling factors, namely the upregulated H_2_O_2_ in mitochondria, photogenerated Fe^2+^ and photoacidification synergistically reinforce oxidative stress via Fenton reactions. Last but not least, intratumoral photoacidification reinforces oxidative stress and simultaneously induces mitochondrial calcium overload, and these two resulting effects with quiet distinct cellular mechanisms synergistically dictates mitochondrial dysfunction and therefore tumor cell death. The high-performance therapeutic efficacy of FMUP based on the abovementioned NIR light-triggered dual damage to mitochondria is unequivocally verified in various tumor cell lines in vitro, cell-derived tumor xenograft (CDX) and patient-derived tumor xenograft (PDX) models in vivo, proclaiming its potential as a safe and potent antitumor agent based on efficiently amplified mitochondrial damage.

## Results

### Characterization of FMUP nanoagents

**Fig. 2:**
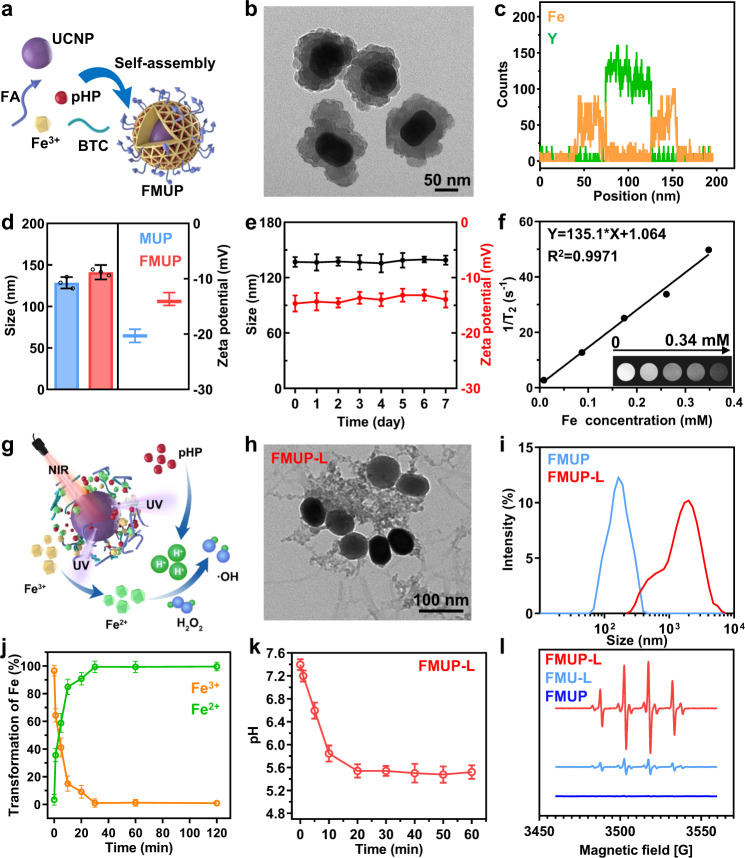
Characterizations of FMUP. **a** Schematic illustration of the construction of FMUP by one-pot self-assembly strategy. **b** TEM image of FMUP. **c** The distribution of Fe element and Y element in FMUP. **d** Size and zeta potential of FMUP and MUP dispersed in water. **e** The stability of FMUP in cell culture medium. **f** T_2_ relaxation rate (1/T_2_, s^−1^) linearity curve and corresponding T_2_-weighted MRI images of FMUP with different Fe concentrations. **g** Schematic illustration of nanoagents in response to NIR light irradiation. **h** TEM image of FMUP after NIR irradiation (980 nm, 1.0 W/cm^2^, 5 min). **i** Size distribution of FMUP dispersed in water before and after NIR irradiation (980 nm, 1.0 W/cm^2^, 5 min). **j** The transformation of Fe^3+^ to Fe^2+^ in FMUP upon continuous NIR irradiation (980 nm, 1.0 W/cm^2^). **k** Evolution of pH value of the FMUP suspension sample in PBS upon continuous NIR irradiation (980 nm, 1.0 W/cm^2^). **l** ESR spectra of various groups. The acid microenvironment could accelerate the Fenton reactions for generating •OH species. Data in (**d**), (**e**), (**j**), and (**k**) were represented as mean values ± SD, *n* = 3 biologically independent samples in (**d**), (**e**), (**j**), and (**k**). A representative image of three biologically independent samples from each group is shown in (**b**), (**c**), (**f**), (**h**), (**i**), and (**l**).

The target nanoagent (denoted as FMUP hereafter) was fabricated via the self-assembly of MOFs (MIL-100, denoted as M for short) shell around the upconversion nanoparticles (UCNPs) core (denoted as U for short) according to the literature procedure with minor modifications (Fig. 2a)^25^. The MOFs shell was constructed using Fe^3+^ as central metal ion while 1, 3, 5-benzenetricarboxylic acid (BTC) and a small portion of FA (denoted as F for short) as bridging ligands. Additionally, a photoacid generator (pHP, denoted as P for short) moiety, namely 4-(2-(4-hydroxy-3, 5-dimethoxyphenyl)-2-oxoethoxy)-4-oxobutan-1-aminium, was encapsulated into the cavities of the MOFs units (Supplementary Fig. 1). The as-prepared nanoagents exhibited an average diameter of ~130 nm and a typical core–shell structure featuring the UCNP component located at the core cloaked by the MOF shell (Fig. 2b). This unique structure was further confirmed by the line scanning (Fig. 2c) and energy dispersive X-ray data obtained from the elemental mapping of FMUP (Supplementary Fig. 2). Owing to the partial participation of FA in chelation with Fe^3+^ ions (Supplementary Fig. 3), the folate ligands were successfully doped, which could be verified by the appreciable influence on the size and surface properties (Fig. 2d). Specifically, FMUP displayed a slightly increased size and decreased zeta potential compared to the pristine MUP, which can be attributed to the decoration of the FA moiety onto the surface of the MOF nanostructure. In terms of pHP, the loading amount was calculated to be up to 12.61%wt (Supplementary Fig. 4).

With FMUP in hand, we further tested its stability. FMUP did not display obvious changes in hydrodynamic radius or zeta potential during 1 week of storage in cell culture medium without fetal bovine serum (FBS), suggesting its favorable stability for in vivo use (Fig. 2e and Supplementary Fig. 5). We also estimated the potential of such Fe-containing nanoagents as T_2_-weighted magnetic resonance imaging (MRI) contrast agents. As illustrated in Fig. 2f, the relaxation rate (1/T_2_) of water protons markedly increased from approximately 2.7 to 49.7 s^−1^, and the gray level of the T_2_-weighted images was significantly augmented upon increasing the concentration of Fe from 0.008 to 0.348 mM, indicating the sufficiently strong magnetism of FMUP for in vivo MRI use and its potential for image-guided anticancer therapy.

### Response capability of FMUP to NIR light

As mentioned above, the design rationale for the nanoagent is based on the UCNP-mediated NIR-to-UV excitation energy conversion^26–29^ and subsequent UV light-mediated reduction of Fe^3+^ to Fe^2+^ and release of H^+^ from the photoacid generator component (Fig. 2g). Upon irradiation with NIR light (980 nm, 1.0 W/cm^2^, 5 min), we clearly observed that the FMUP-dispersed aqueous sample displayed strong emission with a peak at approximately 365 nm due to the encapsulated UCNP (Supplementary Fig. 6). Moreover, the MOF-based core–shell nanostructures obviously disintegrated and aggregated in FMUP-L (FMUP with laser irradiation) group (Fig. 2h), resulting in apparent increase in the size of the nanostructure with a much wider distribution (Fig. 2i). Such change could be assigned to the structural collapse of MOFs units in FMUP originating from the UCNPs-enabled NIR-to-UV excitation energy upconversion and the subsequent UV light-mediated Fe^3+^-to-Fe^2+^ reduction, in which a photochemically induced electron transfer process from a complexing organic ligand to an oxidized metal was involved^30,31^. Specifically, such reduction of central metal ions typically leads to metal-linker bond breaking and hence incorporation of framework defects into MOFs unit, which gives rise to the eventual network collapse of MOFs nanostructure^32–34^. Following the collapse of MOFs units, phase separation of the inorganic UCNPs moiety and the organic linkers was expected to occur, which resulted in the formation of aggregates due to the hydrophobic interactions between the linker units in aqueous milieu. For verification, the evolution of Fe^3+^ in the FMUP-dispersed aqueous sample under NIR light irradiation was evaluated. As shown in Fig. 2j, the evolution of both Fe^3+^ and Fe^2+^ content within the composite nanostructures as a function of NIR light irradiation time clearly indicates the increase in Fe^2+^ ions at the expense of Fe^3+^. Notably, the contents of Fe^3+^ and Fe^2+^ mirror each other, with a decrease in Fe^3+^ that is proportional to the increase in Fe^2+^, suggesting a clean photocatalyzed one-to-one conversion with high efficiency.

In addition to Fe^2+^ release, another outcome of the NIR-to-UV conversion was the photocatalysis of pHP to acidify the microenvironment^35,36^. For verification, we evaluated the acidity evolution of the FMUP suspension upon irradiation with NIR light (980 nm, 1.0 W/cm^2^) using a SNARF^®^-1 probe. As shown in Fig. 2k, the pH of the sample rapidly decreased from weak alkalinity to weak acidity. Together with the released Fe^2+^, such acidification could significantly accelerate the Fenton reaction. To test this aspect, we used 5,5-dimethyl-1-pyrroline-oxide (DMPO) as a •OH trapping agent for ESR spectral characterization (Fig. 2l). The yield of •OH in the FMUP-L group was much higher than that in the FMU-L (the counterpart without photoacid generator) and FMUP groups, again demonstrating the synergistic cooperation between photocontrolled release of Fe^2+^ and photoacidification to reinforce •OH production. Similar results were also obtained via a bleaching experiment with methylene blue (MB) (Supplementary Fig. 7). These results together indicated the good ability of FMUP to respond to NIR light as well as the favorable outcomes for •OH production.

### Intracellular fate of FMUP

**Fig. 3:**
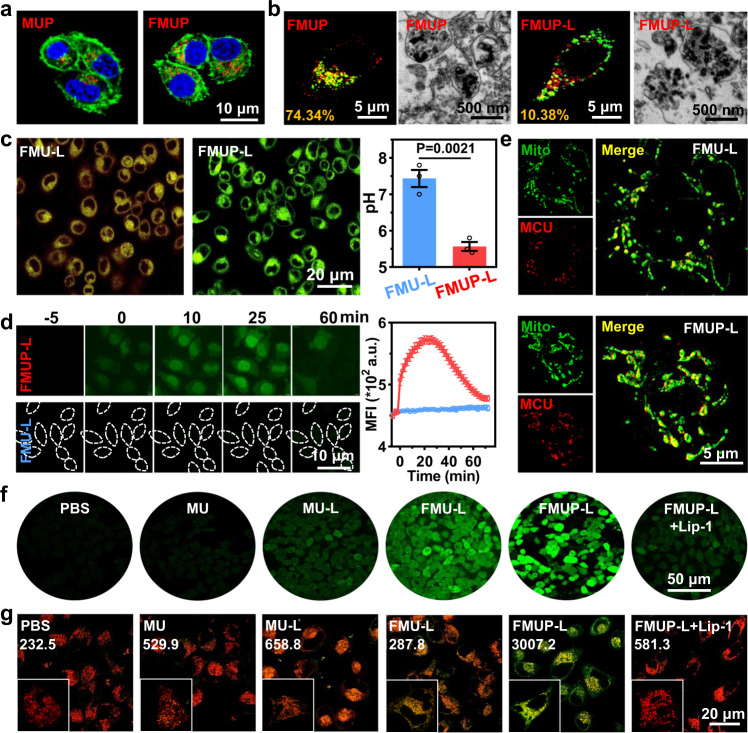
In vitro assessment of the effect of CDT/acidification synergism on mitochondria. **a** CLSM images of MUP and FMUP in HeLa cells. Green: cell membrane; Red: MOFs; Blue: nuclei. **b** CLSM images and TEM images of lysosomal escape of different treatments (FMU-L and FMUP-L). Green: lysosome; Red: MOFs. The number in the lower left corner represents the co-localization rate. **c** CLSM images and further quantitative analysis of pH value in different groups (FMU-L and FMUP-L). The greener the cell, the more acidic it is. **d** CLSM images of calcium influx in group of FMU-L and FMUP-L and corresponding quantitative analysis. Green: Ca^2+^. The outline of dotted line represents the cell. **e** CLSM images of the expression of mitochondrial calcium uniporter (MCU) in the mitochondria. The higher the expression of MCU, the stronger the calcium overload in mitochondria. Green: mitochondrial; Red: MCU. **f** Detection of ROS production in HeLa cytoplasm by DCFH-DA with different treatments. Green: ROS. **g** Detection of lipid peroxidation in HeLa cells by Liperfluo with different treatments. The statistical value in the figure represents the level of lipid peroxidation. Green: lipid peroxidation; Red: mitochondria. Data in (**c**) and (**d**) were represented as mean values ± SD, *n* = 3 biologically independent samples in (**c**) and (**d**). A representative image of three biologically independent samples from each group is shown in (**a**–**g**). *P* value was calculated by the two-tailed Student’s *t* test in (**c**).

Owing to the overexpressed folate receptor (FR) on the tumor membrane^37^ (Supplementary Figs. 8, 9), functionalization of carriers with FA moieties has been proven to be very effective for circumventing the problem of low targeting efficiency to the tumor site. To evaluate the FA-enabled targeting performance, HeLa cells were incubated with Cy5-labeled FMUP (with FA) or MUP (the counterpart without FA), and confocal laser scanning microscopy (CLSM) images were captured. As expected, HeLa cells treated with FMUP presented much stronger red fluorescence than cells treated with MUP (Fig. 3a). These cells were also analyzed by flow cytometry for quantification (Supplementary Figs. 10, 11), and the internalization amount of FMUP was ~2.9 folds than that of MUP, again verifying that decoration with the folate moiety significantly improved internalization by HeLa cells. Such a superior uptake was found significantly inhibited in HeLa cells with downregulated FR or blocked FR (Supplementary Fig. 12), indicating the vital role of FR for FMUP uptake.

After internalization, most foreign nanocarriers are restricted to mature lysosomes. However, ideal mitochondria damage-targeting potency is expected if the Fenton reaction occurs in close proximity to the mitochondria because of the upregulation of H_2_O_2_ in the mitochondria and the extremely short diffusion distance of the •OH species^38,39^. To test whether our FMUP could break through the lysosomal compartmentalization, we evaluated the co-localization of FMUP (labeled with Cy5) and lysosomes (labeled with LysoTracker™ Green) inside HeLa cells (Fig. 3b). Specifically, the co-localization rate of FMUP with lysosomes dramatically decreased from 70.34% to 10.38% upon irradiation with NIR light (980 nm, 1.0 W/cm^2^, 5 min). Moreover, the TEM images provided direct evidence that FMUP resided in lysosomes, while lysosome deconstruction was observed in the FMUP-L group. Such typical lysosomal escape could be plausibly assigned to the high osmotic pressure originating from the sharp upregulation of Fe^2+^ and H^+^ in the lysosomes that FMUP imparted upon NIR light stimulus^40^.

### Intracellular acidification and the dual mitochondrial effects

Next, the capability of the FMUP nanoagents to cause intracellular acidification was evaluated using SNARF^®^-1 as a pH probe (Fig. 3c). Owing to the efficiently generated H^+^ from pHP upon NIR irradiation, HeLa with internalized FMUP emitted vivid green fluorescence after NIR irradiation, indicating an acidified cytoplasm. By using FMU-L as a control, we observed that the fluorescence of HeLa cells did not change to green due to the absence of the photoacid generator pHP. For detailed quantification, the intracellular pH after different treatments was determined from the calibration curves constructed earlier from the CLSM analysis. Notably, the intracellular pH value in the FMUP-L group appreciably decreased to ~5.5 but was still approximately neutral in the FMU-L group, further verifying the crucial role of the photoacid generator for the desired intracellular acidification.

Given that abnormal intracellular acidity generally leads to an imbalance in intracellular Ca^2+^ ^41,42^, we continued to investigate the evolution of intracellular Ca^2+^ levels in HeLa cells using Fluo-3 as a fluorescent calcium probe (Fig. 3d). When the cells were incubated with FMUP or FMU, no noticeable increase in intracellular Ca^2+^ levels in cells prior to NIR light irradiation was observed. In sharp contrast, 5-min irradiation of NIR light unequivocally generated a difference in cytoplasmic Ca^2+^ levels with the probe in FMUP-internalized cells displaying clearly stronger fluorescence than that in FMU-internalized cells and the discrepancy in fluorescence brightness in two cases appreciably increasing up to a maximum level ~25 min after the irradiation. As a result of such photoacidification-enabled upregulation of intracellular Ca^2+^ level, increase in the mitochondrial Ca^2+^ level was also observed. Specifically, it was found that the FMUP-L group clearly displayed much higher expression level of mitochondrial calcium uniporter (MCU)^43–45^ than that of the FMU-L group (Fig. 3e).

In addition to calcium overload in the mitochondria, the observed NIR-triggered release of Fe^2+^ and H^+^ also prompted us to investigate the influence on intracellular •OH generation by using 2′,7′-dichlorofluorescin diacetate (DCFH-DA) as a probe (Fig. 3f and Supplementary Fig. 13a). Considering that multiple enabling factors are involved in the Fenton reaction, we investigated a series of treatment formulations, including PBS, MU (UCNP encapsulated in MIL-100), MU-L (MU with laser irradiation), FMU-L, FMUP-L, FMUP-L + Lip-1 (FMUP-L with Lip-1 pretreatment), for comparison. Compared to the MU group with almost no fluorescence, the cells in the MU-L group exhibited weak fluorescence, which verified the NIR-assisted reduction of Fe^3+^ to Fe^2+^ for the improved Fenton reaction. Owing to the increase in internalization mediated by the FA ligand, a further increased fluorescence, which indicated intracellular •OH concentration, was observed in the FMU-L group. Upon further photoacidification by pHP involvement, cells in the FMUP-L group indeed exhibited the brightest fluorescence, indicating a larger amount of •OH source from the effective Fenton reaction.

Considering the abundant H_2_O_2_ in the mitochondria^18,19^, we also speculated above discrepancy in mitochondria. For verification, we labeled mitochondria with MitoTracker® Red (red color) and evaluated beaconing lipid peroxidation with a Liperfluo probe. As shown in Fig. 3g and Supplementary Fig. 13b, the evolution trend of mitochondria lipid peroxidation level in various cases displayed similar characteristic to that demonstrated in the results regarding •OH production illustrated in Fig. 3f, with the FMUP-L group again showing the most lipid peroxidation in the mitochondria amongst all of the groups. Note that the addition of Lip-1, serving as a ROS scavenger^46,47^, alleviated lipid peroxidation in FMUP-L group, again indicating the role of •OH production in the cell oxidative stress.

### Synergistic reinforcement of mitochondrial damage

**Fig. 4:**
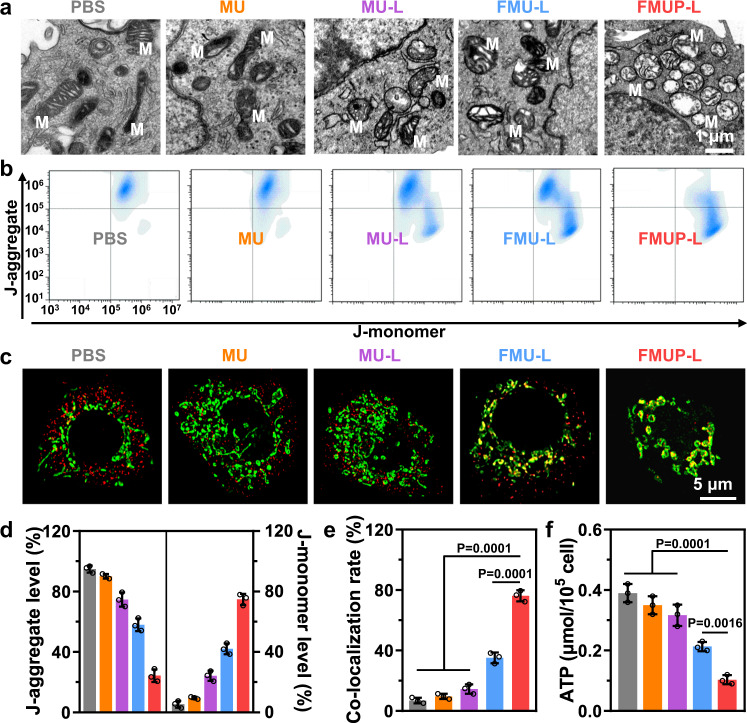
In vitro evaluation of mitochondrial damage by the synergism of CDT and photoacidification. **a** TEM ultrathin sectioning images of mitochondrial integrity in different groups. M in images represents the mitochondria. **b** Mitochondrial membrane potential analysis of HeLa cells treated with different groups by using flow cytometry. **c** CLSM images of cyclophilin D (Cyp D) expression in HeLa cells with different groups. Green: mitochondria; Red: Cyp D. **d** Quantitative analysis of J-aggregate and J-monomer in various treatments. **e** The co-localization rate of nanoagents with mitochondria in different groups. **f** Quantitative analysis of intracellular ATP concentration in various treatments. Data in (**d**–**f**) were represented as mean values ± SD, *n* = 3 biologically independent samples in (**d**–**f**). A representative image of three biologically independent samples from each group is shown in (**a**–**c**). *P* values were calculated by using one-way ANOVA. in (**e**, **f**).

Having demonstrated the dual effects in terms of calcium overload and •OH production in mitochondria, we moved on to investigate their synergistic outcomes on mitochondrial damage. To provide intuitional evidence, we started the investigation with TEM to reveal the change in morphology of HeLa cells after different treatments (Fig. 4a). It was found that cells after treatment of inert MU displayed typical mitochondrial morphology similar to that of cells in the PBS group. In sharp contrast, other formulations of treatment with the involvement of the abovementioned enabling factors unequivocally gave rise to distinguishable morphological changes to the mitochondrial microstructure including profound swelling, outer membrane rupture and crista dissolution with the extent of mitochondria deconstruction augmented in the sequence of MU-L, FMU-L, and FMUP-L.

Considering that mitochondrial membrane potential (MMP) is typically sensitive to both calcium overload and the accumulation of ROS in mitochondria^48^, we next evaluated the MMP of HeLa cells after different treatments by using JC-1 as an indicator, which typically forms aggregates on normal mitochondrial membranes with a relatively high MMP while residing as monomers on the abnormal mitochondrial membrane with a low MMP. According to the flow cytometry data shown in Fig. 4b, d and Supplementary Fig. 14, the FMUP-L group showed the lowest proportion of aggregates (28.8%) and the highest proportion of monomers (70.7%) compared to the other groups, indicating the abnormality of MMP.

In addition to MMP, mitochondrial abnormalities can also be verified by the translocation of the cyclophilin D (Cyp D) protein from the cytoplasm to mitochondria, which is associated with the formation of the mitochondrial permeability transition pore complex^49,50^. To this end, we labeled mitochondria with MitoTracker^®^ Green (green color) and evaluated the translocation of immunostained Cyp D (red color). As expected, most of the Cyp D molecules had transferred to the mitochondria (Fig. 4c), and their co-localization rate was up to 76.71% (Fig. 4e) in the FMUP-L group, which was substantially higher than that of the other groups. Given that mitochondria serve as powerhouses in eukaryotic cells, we also detected ATP production to examine mitochondrial function. Compared with the PBS and MU groups, ATP productivity gradually decreased in the other advanced treatments, with the value decreasing to 0.1 in the FMUP-L group (Fig. 4f). Beyond doubt, these results unequivocally confirmed the most potent mitochondrial damage was induced by FMUP-L, which was ascribed to not only the improved uptake but also the synergism of calcium overload and ROS production.

### In vitro evaluation of cytotoxicity

**Fig. 5:**
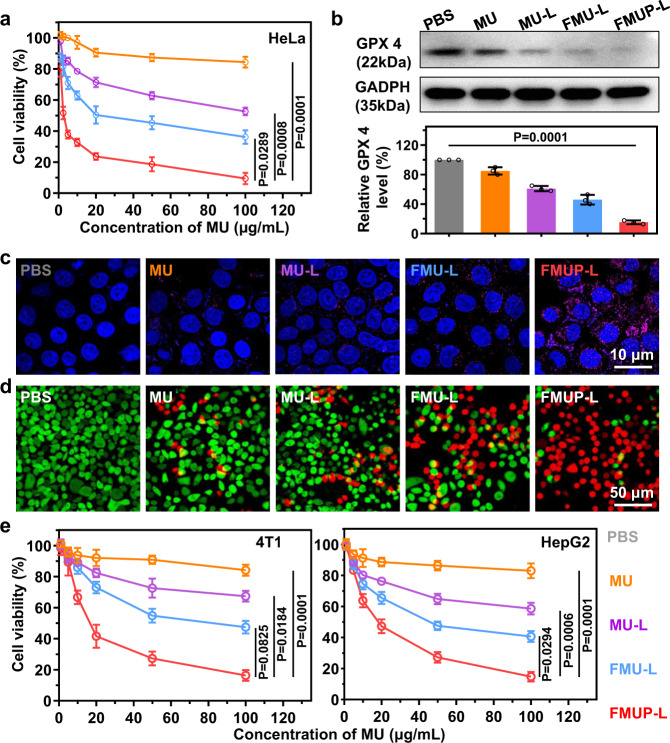
The cytotoxicity of various treatments and the analysis of combined ferroptosis and apoptosis. **a** CCK-8 cytotoxicity analysis of HeLa cells treated with different formulations. **b** Western blotting analysis of glutathione peroxidase (GPX4) and GADPH. The samples derived from the same experiment and that blots were processed in parallel. **c** The analysis of caspase-3 activity in HeLa cells using Alexa 647-conjugated caspase-3 antibody. Pink: caspase-3; Blue: nuclei. **d** Live/dead cytotoxicity analysis of HeLa cells treated with different formulations. Green: live cells; Red: dead cells. **e** CCK-8 cytotoxicity analysis of 4T1 and HepG2 cells treated with different formulations. Data in (**a**), (**b**), and (**e**) were represented as mean values ± SD, *n* = 3 biologically independent samples in (**a**) and (**b**), *n* = 4 biologically independent samples in (**e**). A representative image of three biologically independent samples from each group is shown in (**b**–**d**). *P* values were calculated by using one-way ANOVA in (**a**), (**b**), and (**e**).

Encouraged by the abovementioned results regarding mitochondrial damage, we subsequently investigated the viability of HeLa cells in vitro after different treatments (Fig. 5a). All formulations exhibited a dose-dependent effect on the viability of HeLa cells, and the cytotoxicity increased in the following sequence: MU, MU-L, FMU-L, and FMUP-L. Taking the dose of 100 μg/mL as an example, less than 10% of cells survived in the FMUP-L group, while the cell viabilities in the other groups ranged from ~40% to ~90%. Given that two pathways were involved in mitochondrial damage, the possibility of ferroptosis induced by ROS production and apoptosis induced by calcium overload thus coexisted. To this end, we next investigated the two aspects of ferroptosis and apoptosis.

Ferroptosis was verified by the expression level of glutathione peroxidase 4 (GPX4), which has been regarded as the major indicator of oxidative stress due to its reduction capacity to reactive oxygen species (ROS)^51,52^. As the western blot data show in Fig. 5b and Supplementary Fig. 15, cells in MU group displayed GPX4 level roughly similar to the counterpart in the PBS group due to the inert activity of MU in cells. Upon NIR-assisted reduction of Fe^3+^ to Fe^2+^, the improved Fenton reaction and •OH production definitely decreased the expression level of GPX4 (MU-L group) while the increase in internalization of nanoagents resulted in further suppression of GPX4 expression (FMU-L group). Particularly noteworthy was the involvement of pHP capable of photoacidification that significantly improved the efficiency of Fenton reaction and enabled highly efficient production of •OH (FMUP-L group), which substantially inhibited the expression of GPX4 and consequently presented GPX4 level merely one-tenth of that in the PBS group. Subsequently, the level of intracellular caspase-3, a typical indicator of apoptosis^53^, was evaluated via immunostaining strategy with the results illustrated in Fig. 5c. It can be clearly seen that cells in the FMUP-L group displayed much higher level of caspase-3 as compared to the counterpart level in other groups, indicating the significant involvement of apoptosis in the former that was assigned to the photoacidification effect. Such cooperation of ferroptosis and apoptosis for cytotoxicity in the FMUP-L group was also verified by a live/dead assay (Fig. 5d). Compared with the other treatments, dead cells (red) dominated the cell population in the FMUP-L group, indicating that most cells had been killed due to the combination of ferroptosis and apoptosis. Similar cytotoxic results were also observed in human liver carcinoma cells (HepG2) and mouse breast cancer cells (4T1) in Fig. 5e, and FMUP-L still substantially outperformed the other counterparts.

### In vivo evaluation of biodistribution

**Fig. 6:**
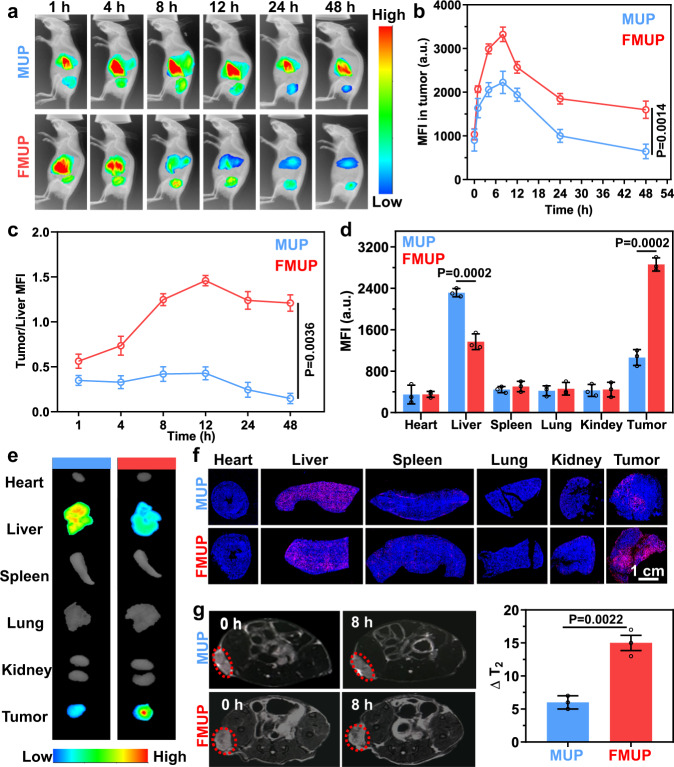
In vivo biodistribution of MOF-based nanoagents. **a** In vivo fluorescence imaging of biodistribution of MUP and FMUP in HeLa tumor-bearing mice over time. The color gradient represents the range from the minimum mean fluorescence intensity (540) to the maximum fluorescence intensity (4600). **b** The mean fluorescence intensity of tumors in the group of MUP and FMUP with elapsing time. **c** The tumor/liver fluorescence intensity ratios in the group of MUP and FMUP with elapsing time. **d**, **e** Ex vivo images of tumors and other main organs at 48 h postinjection and corresponding mean fluorescence intensity analysis in the group of MUP and FMUP. The color gradient represents the range from the minimum mean fluorescence intensity (810) to the maximum fluorescence intensity (3300). **f** Frozen section staining images of main organs and tumors in MUP group and FMUP group. Red: Cy 5-lablled MOFs. Blue: nuclei. **g** T_2_-MRI images of mice before and after injection and corresponding analysis of △T_2_ value in the group of MUP and FMUP. Red dotted circles indicate the tumors. Data in (**b**–**d**, **g**) were represented as mean values ± SD, *n* = 3 biologically independent samples in (**b**–**d**, **g**). A representative image of three biologically independent samples from each group is shown in (**a**, **e**, **f**, **g**). *P* values were calculated by the two-tailed Student’s *t* test in (**b**, **d**, **g**).

It is well documented that the ultimate therapeutic efficacy of anticancer nanomedicines critically depends on their in vivo fate. Taking this into account and being inspired by the aforementioned results, we assessed the fate of the engineered nanoagents in vivo. Considering that the FA ligand is highly correlated with targeting capacity, we enlisted FMUP and MUP for comparison, which were labeled by loading Cy7 dye into the MOF cavity. As the in vivo imaging data in Fig. 6a show, after intravenous injection, the majority of the MUP signals were rapidly enriched in the liver, with a minority of the MUP signals accumulating in the tumor site. In contrast, the fluorescence signal of FMUP at the tumor site increased substantially at the expense of the signal in the liver over time, indicating the pivotal role of doped FA for tumor accumulation. Despite this discrepancy, the accumulation of these two types of model agents at the tumor site peaked at 8 h (Fig. 6b). Upon further calculation of the signal ratio of the tumor/liver, we observed a magnified discrepancy evolution. Specifically, the ratio in the FMUP group increased and reached a peak value of ~1.5, while the value in the MUP group continuously declined and reached 0.2 in 48 h (Fig. 6c).

To gain deeper insight, we excised the main organs, detected the fluorescence, and quantified the signal intensity. As shown in Fig. 6d, e, the fluorescence intensity of the excised tumor in the FMUP group was 2.4-fold higher than that in the MUP group, unequivocally proclaiming the FA-enabled tumor-targeting capacity of FMUP for improved therapeutic performance. In other organs, the main discrepancy was found in the liver, and the FMUP group exhibited half of the accumulation the MUP group displayed, thus reducing the possibility of side effects to the liver. For higher resolution, these organs were further treated as frozen slices for microscopic observation (Fig. 6f). Compared with the MUP group, the FMUP group displayed substantially improved infiltration in the tumors and significantly suppressed accumulation in the liver. Moreover, ICP-MS analysis results revealed that these nanoagents could be excreted through feces (Supplementary Fig. 16). In addition, the T_2_ signal of Fe^3+^ from our nanoagents also enabled us to investigate the biodistribution via MRI (Fig. 6g). FMUP displayed remarkably stronger contrast than MUP in T_2_ imaging at the tumor sites, again confirming its superior tumor-targeting capacity.

### In vivo antitumor effects in cell derived xenograft (CDX) model

**Fig. 7:**
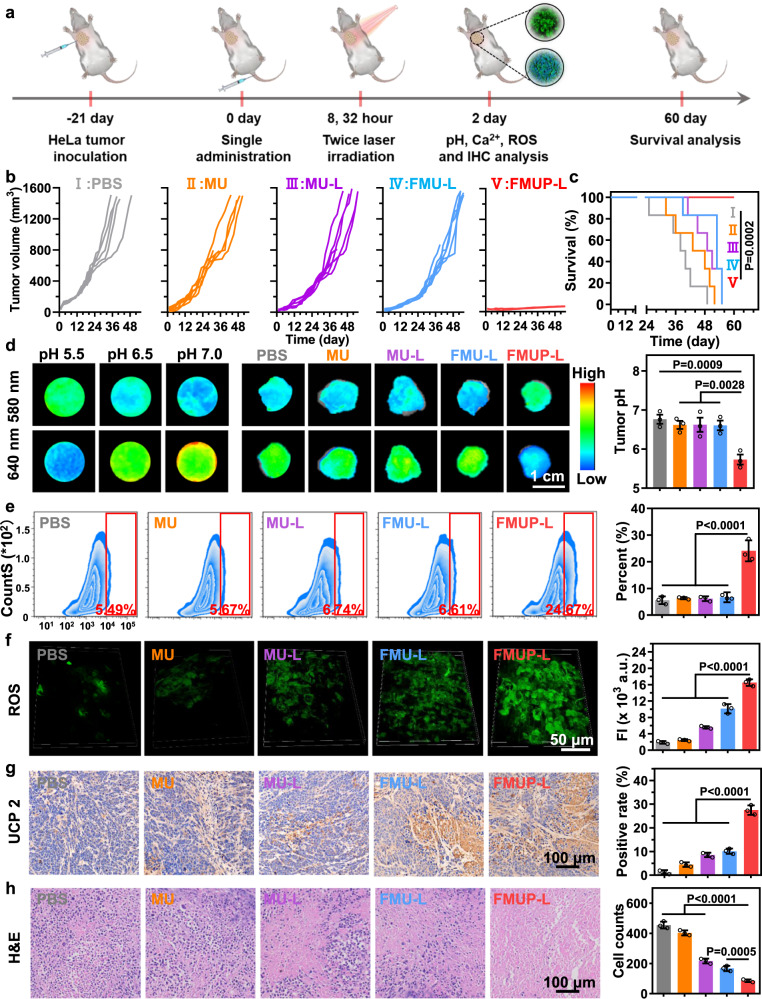
In vivo antitumor effects in HeLa-cell-derived tumor xenograft model. **a** Schematic illustration of anticancer therapy for HeLa primary tumors. Note that mice in the groups with the involvement of light irradiation underwent treatment of irradiation (980 nm, 1.0 W/cm^2^, 5 min) twice, at 8 h and 32 h, respectively, after a single intravenous administration. **b** Tumor growth curves of different treatments over 55 days (*n* = 6). **c** Survival curves of tumor-bearing mice treated with different groups. **d** Fluorescence imaging of tumor pH value in different groups by using SNARF®-1 and corresponding quantification analysis. The color gradient represents the range from the minimum mean fluorescence intensity (530) to the maximum fluorescence intensity (1680). **e** The analysis of calcium influx in tumors with different treatments by using flow cytometry and corresponding analysis. **f** Two-photon fluorescence imaging and corresponding fluorescence intensity analysis of ROS in tumors in different treatments by using DCFH-DA. **g** Immunohistochemical staining images and further quantitative analysis of UCP 2 in tumor tissue for indicating the mitochondrial damage. **h** Hematoxylin and eosin (H&E) staining images of tumors in different groups and corresponding quantitative analysis for tumor cells. Data in (**d**–**h**) were represented as mean values ± SD, *n* = 6 biologically independent samples in (b, c), *n* = 3 biologically independent samples in (**d**–**h**). A representative image of three biologically independent samples from each group is shown in (**d**–**h**). *P* values were calculated by the one-way ANOVA in (**d**–**h**). *P* value was calculated by the Log-rank test in (**b**).

The abovementioned in vivo results presented motivation for further investigation on the in vivo therapeutic performance of our nanoagents in HeLa tumor-bearing mice. For comparison, the HeLa tumor-bearing mice were randomly divided into five groups and treated with the formulation of PBS, MU, MU-L, FMU-L, and FMUP-L, respectively. In a typical protocol, mice in the groups with the involvement of triggering light underwent treatment of laser irradiation (980 nm, 1.0 W/cm^2^, 5 min) twice, at 8 h and 32 h, respectively, after a single intravenous administration (Fig. 7a). Such treatment regimen was based on the time for FMUP nanoagent accumulation peaking after intravenous administration (Fig. 6a), the postirradiation incubation-time-dependent in vitro cytotoxicity (Supplementary Fig. 17a) and the irradiation-time-dependent cytotoxicity of FMUP nanoagents (Supplementary Fig. 17b). As shown in Fig. 7b, compared with the PBS group, MU alone did not exert an appreciable influence on the growth of HeLa cells, due to poor tumor accumulation and low Fenton reaction activity. Similar to the results observed in in vitro cytotoxicity (Fig. 5a), the factors of light irradiation, FA-enabled tumor targeting, and photoacid generator unequivocally played their respective roles in inhibiting the tumors with the consequent tumor size diminished in the sequence of MU-L, FMU-L, and FMUP-L. Specifically, upon NIR light irradiation, photo-reduced Fe^2+^ with a higher Fenton reaction activity appreciably promoted the generation of ROS to kill tumor (MU-L). Moreover, the antitumor effect in FMU-L group was further improved owing to the FA-mediated increase in accumulation of nanoagents at tumor site. Notably, with the additional involvement of the photoacid generator, treating tumor-bearing mice with FMUP-L nearly completely inhibited tumor growth with a 100% survival rate after 60 days (Fig. 7c), which could be attributed to efficient tumor targeting as well as the combined effects of efficient CDT and calcium overload-mediated mitochondrial damage. In contrast, all mice in the other groups gradually died within 55 days.

Taking the synergistically reinforced mitochondrial damage for in vitro cytotoxicity, we evaluated whether this aspect was highly correlated with the above distinct therapeutic outcomes. To estimate the ability of regulating the intratumoral acidity, SNARF^®^-1 was used as a probe and administered via intravenous tail injection to map pH fluctuations at the tumor sites on day 14 using an animal fluorescence imaging system (Fig. 7d). Referenced by the calibration curves obtained via the imaging system, the pH value at the tumor site after FMUP-L treatment was found decreased to ~5.6, while the counterparts in other groups remained at ~6.6, highlighting the pivotal role of the photoacid generator in intratumoral acidification. Consequently, the intratumoral Ca^2+^ level (detected by the Fluo-3 probe) in the FMUP-L group was significantly higher than that in the other groups without the involvement of a photoacid generator (Fig. 7e and Supplementary Fig. 18), laying the foundation for FMUP-L treatment to enable calcium overload in tumor cells.

To verify the promoting effect of intratumoral acidification on Fenton reaction, we evaluated the ROS production at the tumor site via two-photon fluorescence microscopy using DCFH-DA as a probe. With the involvement of other promoting factors including the NIR-assisted reduction of Fe^3+^ to Fe^2+^ and the FA-mediated improvement in internalization, the ROS productivity was found increased in the sequence of MU, MU-L, FMU-L, and FMUP-L (Fig. 7f). Due to the synergism of the above calcium overload and ROS production, the expression level of uncoupling protein 2 (UCP 2, a typical indicator of mitochondrial dysfunction^54,55^) in the FMUP-L group was markedly stronger than that in the other groups (Fig. 7g), indicating the most substantial mitochondrial damage in tumor cells in vivo. The FMUP-L group also displayed the obvious necrosis in the H&E slice imaging results (Fig. 7h). It also deserves mentioning that the potent antitumor effects of FMUP-L were achieved with few abnormalities to the histology of the main organs (Supplementary Fig. 19), serum biochemical indicators (Supplementary Fig. 20), and body weight (Supplementary Fig. 21), indicating the good safety of our nanoagents.

### In vivo antitumor effects in patient-derived tumor xenograft (PDX) model

**Fig. 8:**
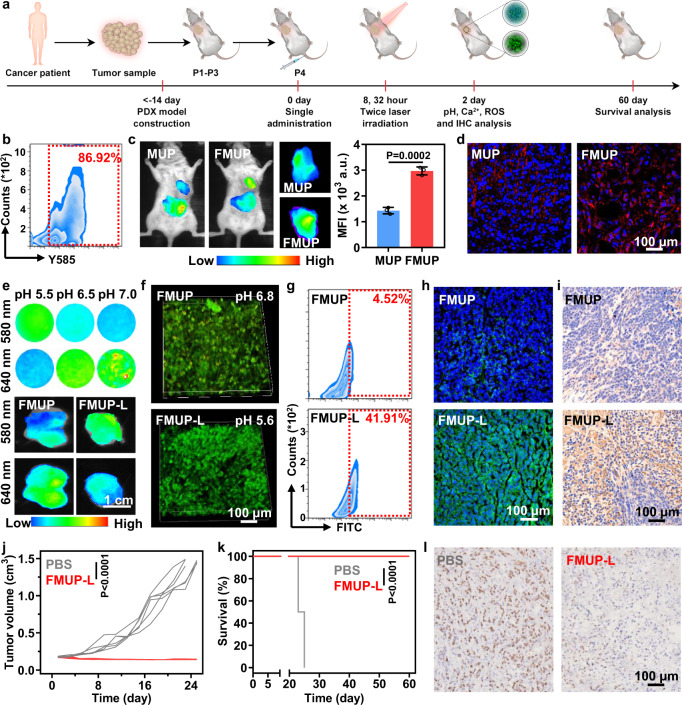
In vivo antitumor effects in patient-derived tumor xenograft (PDX) model. **a** Schematic illustration of procedure for evaluating antitumor activity in liver-cancer-PDX model. Note that mice in the groups with the involvement of light irradiation underwent treatment of irradiation (980 nm, 1.0 W/cm^2^, 5 min) twice, at 8 h and 32 h, respectively, after a single intravenous administration. **b** The analysis of the expression of folate receptor in human hepatoma cells sourced from PDX tumor by using flow cytometry. **c** In vivo fluorescence imaging of biodistribution of MUP and FMUP in tumor-bearing mice 8 h after injection and the corresponding quantitative analysis for various tumors. The color gradient represents the range from the minimum mean fluorescence intensity (1400) to the maximum fluorescence intensity (4200). **d** Frozen section staining images of tumors in the group of MUP and FMUP. Red: Cy 5-lablled MOFs; Blue: nuclei. **e** Fluorescence imaging of tumor pH in the group of MUP and FMUP using SNARF®-1. The color gradient represents the range from the minimum mean fluorescence intensity (560) to the maximum fluorescence intensity (1830). **f** Two-photon fluorescence imaging of tumor pH in the group of FMUP and FMUP-L using SNARF^®^-1. **g** The quantitative analysis of ROS in tumors in the group of FMUP and FMUP-L via flow cytometry using DCFH-DA as the probe. **h** Frozen section staining imaging of tumors for evaluating the calcium influx after different treatments using Fluo-3 as the probe. **i** Immunohistochemical staining images and further quantitative analysis of UCP 2 in tumor tissue for indicating the mitochondrial damage. **j** Tumor growth curves of different treatments over 25 days. **k** Survival curves of tumor-bearing mice in the group of PBS and FMUP-L. **l** Representative cell proliferation analysis of tumor tissue by Ki 67 method. Data in (**c**), (**j**), and (**k**) were represented as mean values ± SD, *n* = 3 biologically independent samples in (**c**), *n* = 6 biologically independent samples in (**j**, **k**). A representative image of three biologically independent samples from each group is shown in (**b**–**i**, **l**). *P* value was calculated by the two-tailed Student’s *t* test in (**c**, **j**). *P* value was calculated by the Log-rank test in (**k**).

Cell lines widely used in routine antitumor experiments typically suffer from the lack of strong clinical relevance. As practical alternative models, PDX models mostly retain the principal histological and genetic characteristics of their donor tumor and remain stable across passages, which make them more reliable for clinical outcome prediction and preclinical drug evaluation. To confirm the clinical applicability of our nanoagent, we evaluated its therapeutic effect in a PDX model, which was established by transplanting a primary tumor sample resected from a liver-cancer patient into the axilla of NOD-SCID mice followed by engraftment three times for subsequent use (Fig. 8a). Based on flow cytometry analysis of dissociated cells, we confirmed that the high expression of the FA receptor remained in the PDX model (Fig. 8b). Additionally, FMUP (labeled by Cy7) showed ~2.4-fold accumulation in tumors (Fig. 8c) compared with its counterpart without FA functionalization. This FA-mediated targeting capacity was further echoed at the histological level, with far more nanoagents observed in the tumor slice in the FMUP group than those in the MUP group (Fig. 8d).

Having confirmed the targeting capacity of FMUP in the PDX model, we continued to investigate the therapeutic outcomes of FMUP-L treatment. To this end, patient-derived tumor-bearing mice were treated using 980-nm laser (980 nm, 1.0 W/cm^2^, 5 min) twice, at 8 h and 32 h, respectively, after a single intravenous administration of FMUP nanoagent. Both in vivo imaging and two-photon microscopy imaging of tumors with pretreatment of SNARF^®^-1 probe unequivocally confirmed that the pH value at the tumor site in the FMUP-L group was lower than that of the FMUP group (Fig. 8e, f). Downstream of intratumoral acidification was further evaluated by flow cytometry and frozen section staining imaging. Specifically, the FMUP-L group showed a substantially shifted beaconing signal of DCFH-DA probe (Fig. 8g and Supplementary Fig. 22) and a stronger signal of Fluo-3 (Fig. 8h) as compared to those in FMUP group, therefore strengthening the argument that intratumoral acidification could induce both calcium overload and efficient ROS production. Owing to the combination of these two enabling factors, significantly elevated expression of UCP 2 was observed on FMUP-L-treated tumor slices (Fig. 8i). Such potent mitochondrial damage resulted in almost complete inhibition of tumor growth (Fig. 8j) and achieved a 100% survival rate after 60 days, in sharp contrast to the outcome that all untreated mice died within 25 days (Fig. 8k). Moreover, a prominent suppression effect on tumor proliferation (Fig. 8l) was clearly observed in mice of the FMUP-L group as compared to those in the PBS group. These results together suggested that our FMUP-L nanoagent offered specific and highly effective antitumor therapy in the PDX model, thus shedding light on its potential clinical efficacy.

## Discussion

Among emerging antitumor strategies explored for addressing long-standing issues that the traditional single-modality strategies generally encounter, multiple-approach-involved antitumor strategies especially those with synergistic effects hold great potential for improved therapeutic efficacy. Owing to the vital physiological roles of mitochondria, which is essential for cell survival and proliferation, strategies capable of activating multilevel mitochondrial damage pathways via spatiotemporally controllable manner are particularly promising for antitumor treatments with desired precision and enhanced therapeutic index. In this work, we presented a type of MOFs-based FMUP nanoagent that enables NIR light-triggered two distinct mechanisms, namely oxidative stress based on Fenton reactions and calcium overload originating from intracellular acidification, with both of them contributing to the mitochondria-dysfunction-associated eradication of tumor cells.

Such dual-mitochondria-damage capacity of MOFs-based nanoagent was derived from the full exploitation of functional components in FMUP nanoagents. Specifically, the UCNPs-enabled NIR light-triggering mode permitted the controlled action of the FMUP in the deep tissue-penetrable optical window. The manner of simultaneously phototriggering dual mitochondrial damage imparted the spatial-temporal consistency of actions, which is beneficial to boosting the therapeutic efficacy. Of particular note is that the Fenton reaction was synergistically reinforced based on triple enabling factors, namely the upregulated H_2_O_2_ species in mitochondria, the NIR light-mediated in situ release of Fe^2+^ and photoacidification of microenvironment, which markedly strengthened the intratumoral oxidative stress. Additionally, photoacidification acted as the enabling factor in both Fenton reactions and calcium overload, presenting a one-stone-two-birds protocol for triggering the antitumor action. Moreover, the fact that the upregulated H_2_O_2_ in mitochondria acting as the endogenous stimulator for triggering Fenton reactions is expected to confine the range of action locally to the targets and therefore present antitumor capabilities with the desired specificity. In addition to the validation of the cytotoxicity of FMUP in various tumor cell lines and CDX models, the in vivo experiments in PDX models unequivocally indicated that a single administration of FMUP nanoagents with twice irradiation of 980-nm light could completely suppress the tumor development, supporting FMUP-L as a promising modality to fight against cancer.

To the best of our knowledge, such a paradigm where a photoacid generator moiety incorporated in MOFs nanostructure enabling NIR light-triggered dual damage to mitochondria synergistically based on the intratumoral photoacidification process with the involvement of oxidative stress and calcium overload has not been reported previously. This proof-of-concept implementation opens up a way of exploring optically manipulating intracellular acidity/alkalinity for synergistic antitumor therapeutics with enhanced precision and anticancer efficacy. For instance, replacing photoacid with photoalkali components (e.g., malachite green) and exploring the potential effects on cell viability and synergism with other therapeutic modalities could plausibly provide promising opportunities to antitumor applications. Further opportunities also exist in imparting additional and more potent therapeutic activities via the way of optimizing the functional components of MOFs. For example, replacing Fe^3+^ with Cu^2+^ in constructing the MOF shells is expected to enable Cu^+^-mediated pyroptosis pathway and chemodynamic therapy based on the photocatalyzed reduction of Cu^2+^ to highly active Cu^+^ for Fenton-like reaction. Additionally, integrating drug active ingredients into the organic linkers could be a way for introducing additional antitumor activity for further enhancement of therapeutic effects.

## Methods

### Materials

Folic acid (FA, 99.5%), 1,3,5-benzene tricarboxylic acid (BTC, 99.5%), FeCl_3_·6H_2_O, methylene blue (MB, 99.5%) and methanol (99%) were purchased from Sigma-Aldrich (U.S.A.). Penicillin-streptomycin, Dulbecco’s modified eagle medium (DMEM) and phosphate-buffered saline (PBS, pH = 7.4) were purchased from GIBCO BRL (Gaithersburg, MD, U.S.A.). Certified Fetal Bovine Serum, FBS (VivaCell, Shanghai, China). Goat anti-rabbit IgG-Alexa 647, CCK-8, 2′, 7′-Dichlorofluorescin diacetate (DCFH-DA), 5,5′,6,6′-Tetrachloro-1,1′,3,3′-tetraethyl-imidacarbocyanine iodide (JC-1), and Hoechst 33342 were purchased from Beijing Solarbio Science&Technology Co., Ltd. (Beijing, China). LabPAGE 4-20% was purchased from Beijing LABLEAD BIOTECH Co., Ltd. (Beijing, China). Anti-cyclophilin 40 (Cyp D) antibody, anti-mitochondrial calcium uniporter (MCU) antibody, anti-uncoupling protein 2 (UCP 2) antibody, anti-Ki 67 antibody, and anti-glutathione peroxidase 4 antibody were purchased from Abcam (Cambridge, UK). PE anti-FOLR1, purified anti-human folate receptors α and β (FR-αβ) were purchased from DAKEWE (Shenzhen, China). The liproxstatin-1 was purchased from Selleck Chemicals, USA. Alexa Flour 488-phalloidin, LIVE/DEAD™ cell imaging kit, SNARF^®^-1, LysoTracker™ Green DND-26, MitoTracker™ Green, MitoTracker™ Red, Fluo-3, and folate receptor alpha polyclonal antibody were purchased from Life technologies, USA. The fluorescent hydrophilic dyes Cy5 and Cy7 were purchased from Fanbo Biochemical Co., Ltd. (Beijing, China). Liperfluo was purchased from Dojindo Molecular Technology, Inc. The oil dispersible upconversion nanoparticle (NaGdF_4_: 30%Yb, 0.5% Tm @NaYF_4_; Ex: 980 nm, Em: 365 nm; purple light) was purchased from Suzhou Yansheng Bio-tech Co., Ltd. SiRNA (GAUGUUUCCUACCUAUAUAdTdT) was purchased from Sangon Biotech (Shanghai) Co., Ltd. Breast cancer line (4T1), human cervical carcinoma (HeLa), and liver hepatocellular cells (HepG2) were purchased from Beijing Xiehe Hospital, which have been confirmed without mycoplasma contamination by mycoplasma detection kit (Beijing Solarbio Science & Technology Co., Ltd., CA1080). 980-nm laser meter (LWIRL980-10W-F, Beijing) was obtained from Beijing Laserwave Optoelectronics Technology Co., Ltd. (Beijing, China). The intensity of NIR laser used in the all experiments was 1.0 W/cm^2^ and the irradiation time was 5 min for avoiding generating heat in tissue.

### Preparation of photoacid

4-(2-(4-hydroxy-3, 5-dimethoxyphenyl)-2-oxoethoxy)-4-oxobutan-1- aminium was synthesized by the method reported in the literature. Typically, a solution of γ-O-(3,5- dimethoxy-4-hydroxyphenacyl) t-butyl N-t-boc γ-aminobutyric acid (723 mg, 1.45 mmol) in 10 ml trifluoroacetic acid (TFA) was cooled to 0 °C and reacted for 4 h with stirring. The resulting solution was concentrated by a rotary evaporator and residual solvent removal with a high vacuum pump. The crude product was extracted with a solution of H_2_O/EA. The aqueous layer was collected and the water was lyophilized off to give a clear oil of γ- O-(3,5-dimethoxy-4-hydroxyphenacyl) γ-aminobutyric acid, trifluoroacetate salt (650 mg, 98%).

### Preparation of FMUP

FMUP were prepared using the method reported in the literature with minor modifications^25^. In a typical procedure: first, 40 mL of 7.5 mM FeCl_3_ solution (in methanol) and 1.5 mL of UCNP methanol solution (1 mg/mL) were mixed under medium stirring for 30 min. Afterward, 20 mL of 7.5 mM BTC solution (in methanol), 20 mL of 7.5 mM FA solution (in methanol), and 20 mg photoacid pHP were added and mixed with the former solution for stirring about another 30 min.

Next, the mixture was transferred into a 50 °C Oil bath and reacted for 6 h. The reaction product was collected by centrifugation (9800 × *g*, 10 min) and washed 3 times with ethanol and deionized water. Finally, the product was stored for use after drying. To facilitate the subsequent fluorescent imaging and measurement, The FMUP/Cy5 or FMUP/Cy7 could be obtained by mixing Cy5 or Cy7 into the reaction solution in the dark. The unloaded free Cy5 or Cy7 molecules were removed by centrifugation at 9800 × *g* with 10 min (DHS MC22R, China).

### DLS characterization of FMUP

The size and zeta potential of FMUP or MUP were determined using a Malvern laser particle size analyzer with associated software (NANO ZS, England, version 7.12). In order to determine the stability of FMUP, the FMUP were stored in cell culture medium (DMEM) without FBS. Then its size and zeta potential were detected every day. For investigating the size stability of FMUP at different pH, the FMUP was dispersed into FBS-free DMEM with various pH values (5.0, 6.5, and 7.4). The corresponding size was measured every day.

### TEM characterization of FMUP

The FMUP was dropped on the copper grid for three replicates. The morphology of the FMUP was observed by TEM with associated software (JEOL JEM-1400, Japan, version 1.7.18.2349) at 100 kV. In order to analyze the elements proportion of component in FMUP, a mapping scan analysis was performed.

### BET characterization for different formulations

The Brunauer-Emmett-Teller (BET) surface area and pore size of FMU were measured using ASAP2050 system (England).

### MRI characterization of FMUP

MRI of the FMUP solutions with various Fe concentrations was performed using a small animal MRI (9.4 T) scanner with associated software (Bruker, America, version 6.01) for three replicates.

### Characterizations of FMUP before and after NIR light irradiation

The morphological changes of FMUP before and after NIR irradiation (980 nm, 1.0 W/cm^2^, 5 min) were measured by TEM with associated software (JEOL JEM-1400, Japan, version 1.7.18.2349). Meanwhile, DLS was used to determine the size of MOF after NIR irradiation. Correspondingly, we measured the amount of Fe^2+^ sourced from the FMUP. The concentration of Fe^2+^ and Fe^3+^ ions in the FMUP during NIR irradiation (980 nm, 1.0 W/cm^2^) was determined by the standard Fe-detected method. Fe^2+^ can form a stable complex with 1,10-phenanthroline which has a maximum absorbance at 510 nm. The absorbance of the complex was monitored using automatic microplate reader (Tecan Infinite M200, Switzerland) at 25 °C.

### NIR light-triggered pH reversal in vitro

Before measuring the pH value change of solution, the standard solutions with different pH values (5.0, 5.5, 6.0, 6.5, 7.0, and 7.5) were mixed with SNARF^®^-1 at a volume ratio of 1:100, respectively. And the fluorescence intensity at 580 nm and 640 nm of various standard solution was determined by automatic microplate reader. As a result, the calibration curve was obtained. The solution of FMUP mixed with SNARF^®^-1 at a volume ratio of 1:100 before NIR irradiation. In order to investigate change of pH in a buffer environment, the aqueous solution with dispersed FMUP was added into PBS solution and further mixed with SNARF^®^-1. Then, the mixed solutions were both irradiated continuously for 60 min (980 nm, 1.0 W/cm^2^). And fluorescence intensity at 580 nm and 640 nm of the FMUP solution were monitored by automatic microplate reader with associated software (version 1.6.19.2) at room temperature every 5 min. Then, the pH values with different irradiation time were calculated by the calibration curve.

### Evaluating the effect of NIR light-triggered acidification on ROS production in vitro

Before measuring generation of ROS in HeLa cells, the ability of generating more •OH in acidic environment was evaluated and compared by MB bleaching assay. 200 μL mixed solution comprising with MB (10 mg/L) and different formulations (FMUP, FMU-L, and FMUP-L) with equal Fe concentration reacted with H_2_O_2_ (200 μM) in 96-well plate and further were irradiated with various time (0 min, 1 min, 5 min, 10 min, 20 min, 30 min, 40 min, 50 min and 60 min), respectively. The absorption intensity at 664 nm at each time point was measured for determining the ability of the ROS production. Bleaching of MB, due to the presence of •OH in a sample, was indicated by a discoloration from a dark blue color to an almost white color. In addition, the •OH production with various treatments were measured by electron spin-resonance spectroscopy (ESR). In detail, the reaction solution was mixed with 5,5-dimethyl-1-pyrroline N-oxide (DMPO) at a volume ratio of 1:50 before measurement.

### Evaluating the role of FA on internalization by CLSM

For the cellular uptake study, HeLa cells were incubated with different formulations (FMUP/Cy 5 and MUP/Cy5). First, HeLa cells were seeded onto 35 mm glass-bottom dishes at a density of 1 × 10^5^ cells in 1 mL of culture media for adhesion. After 12 h adhesion, 50 μL Cy5-labeled various formulations (1 mg/mL) were added into 1 mL cell culture media. After 12 h internalization, the cells were washed by cold PBS, and then were fixed by 4% paraformaldehyde for 20 min. Next, the cell membrane and nuclei were stained with Alexa Fluor 488-phalloidin (Green) for 30 min at 37 °C and Hoechst 33342 (Blue), respectively. The corresponding fluorescence images were obtained by CLSM with associated software (NIKON, A1, version 5.20.00).

### Evaluating the role of FA on internalization by flow cytometry

Flow cytometry (Beckman Coulter CyAn ADP, USA) was applied to further quantify the cellular uptake in different groups (FMUP/Cy 5 and MUP/Cy5). First, HeLa cells were seeded in 48-well plate for 12 h until cell attached the bottom. Next, the cells were incubated with 0.05 mg/mL Cy5-labeled MOFs for another 12 h. Then cells were washed by cold PBS with three times. Subsequently, the cells were trypsinised and further were collected by centrifugation (500 × *g*, 5 min). Finally, the uptake amounts with different treatments were determined by flow cytometer (FCM) with associated software (version 2.3.1.22). The R673-APCA750 (gain value: 30) was chosen as the fluorescence channel for acquiring the fluorescence intensities of various nanoagent samples. Data were obtained from 15,000 cells for each sample. In order to confirm the effect of endocytosis time on the amount of endocytosis, the same method was executed within different internalized times.

### Evaluating the role of FR on internalization by FCM

Before evaluating the effect of FR on internalization, the FR expression levels of three cell lines were evaluated by FCM. Specifically, the collected cells were incubated with the fluorescent antibody at 4 °C for 30 min (5 μL antibody was added into100 μL suspension). Then, cells were centrifuged (500 × *g*, 5 min) and resuspended in 400 μL staining buffer solution. Before testing, 2 μL 7AAD^+^ was added into solution for distinguishing live and dead cells. The B690 and Y585 (gain value: 30) were chosen as the fluorescence channel.

To confirm that the uptake is indeed mediated by folate receptors, the receptor blocking or silencing experiments were performed with the FR-positive HeLa cells. Firstly, HeLa cells were seeded in 48-well plate for 12 h until cell attached the bottom. In the FR receptor blocking experiment, HeLa cells were first incubated with antibody for 30 min and then 0.05 mg/mL Cy5-labeled FMUP was added in well. In the FR silencing experiment, HeLa cells were first incubated with transfection reagent containing siRNA (5 pmol/well) for 2 days and then 0.05 mg/mL Cy5-labeled FMUP was added in well. As a control, the cells were directly incubated with the FMUP without blocking the FR. After 12 h incubation, cells were washed using cold PBS three times. Subsequently, the cells were trypsinised and collected by centrifugation (500 × *g*, 5 min). Finally, the uptake amounts with different treatments were determined by flow cytometer (FCM) with associated software (version 2.3.1.22). Data were obtained from 15000 cells for each sample.

### Evaluating the effect of NIR light-triggered acidification on lysosome escaping by CLSM

The cultural conditions for HeLa cells were described above and incubated with 0.05 mg/mL Cy5-labeled FMUP for 12 h incubation. Before labeling lysosome, the cells were irradiated by NIR with 5 min (980 nm, 1.0 W/cm^2^), and were put into incubator for 1 h. For comparison, the cells treated with FUMP were not suffered from NIR irradiation. Next, Lyso-Tracker Green at a concentration of 50 nM was added and incubated with cells for 30 min at 37 °C. Before observation, the unreacted dye was removed and the cells were washed by cold PBS. Lyso-Tracker Green was excited at 488 nm and Cy5 in FMUP/Cy5 was excited at 664 nm, respectively. The corresponding fluorescent images at 500–545 nm and 660–710 nm were obtained by CLSM. The co-localization rates of nanoagents and lysosomes in various treatments were determined via the co-localization analysis module in NIS-Elements AR Analysis software (version 5.20.00) of confocal laser scanning microscope (Nikon, A1).

### Evaluating the effect of NIR light-triggered acidification on lysosome escaping by TEM

The HeLa cells were cultured and treated with the same concentration of FMUP (50 μg/mL). After 12 h incubation, cells in FMUP-L group were treated with laser irradiation (980 nm, 1.0 W/cm^2^, 5 min). After that, cells in both groups were incubated for another 0.5 h. Then cells in various groups were fixed with 1 mL general fixative (containing 2.5% glutaraldehyde in 0.1 M Phosphate buffer) at 4 °C for overnight. After dehydration, cells were embedded in epoxy resin and the resin was stored at 55 °C for 48 h to allow resin polymerization. The embedded samples were then sliced with a thickness of 50–70 nm. Finally, the cell sections were stained with 5% uranyl acetate for 15 min and 2% lead citrate for 15 min before TEM imaging. The operating voltage of the TEM was 120 kV and the operating current was 65.10 μA under vacuum. Images of lysosomes were obtained using TEM with associated software (JEOL JEM-1400, Japan, version 1.7.18.2349).

### Assessing NIR light-triggered acidification of HeLa cells by CLSM

The cultural conditions for HeLa cells were described above and FMUP were added and incubated into the cells at 0.05 mg/mL for 12 h. Before NIR irradiation (980 nm, 1.0 W/cm^2^, 5 min), the cells were incubated with pH indicator (SNARF^®^-1) at 50 nM for 30 min. SNARF^®^-1 was excited at 488 nm. The corresponding fluorescent images at 560-590 nm and 620-650 nm were taken by CLSM. And the fluorescence ratio of intensity_580 nm_ and intensity_640 nm_ were calculated by NIKON analyzer. Subsequently, the pH value was calculated by using the calibration curve.

### In vitro assessing the effect of NIR light-triggered acidification on calcium influx by CLSM

The cultural conditions for HeLa cells were described above and FMUP or FMU were added at 0.05 mg/mL for 12 h incubation. Before NIR irradiation, the cells were incubated with Ca^2+^ indicator (Fluo-3) at 5 μM for 30 min. Then, the excess Fluo-3 and MOFs were removed and washed by cold PBS in dish. Finally, the fresh DMEM media were added into the dish. Next, we employed the CLSM to monitor the distribution of Ca^2+^ in HeLa cells (denoted as −5 min). Subsequently, the cells were received NIR irradiation (980 nm, 1.0 W/cm^2^, 5 min) in each group (FMUP-L and FMU-L). Finally, we performed point-in-time imaging and calculated the concentration of calcium in cell with elapsed time.

### In vitro assessing the effect of NIR light-triggered acidification on calcium overload in mitochondrial by CLSM

HeLa cells were seeded and cultured in 35-mm glass-bottom dishes for adhesion. Then the cells were incubated with different nanoagents (FMU and FMUP with identical MU concentration, 50 μg/mL). After 12 h incubation, cells in both groups were treated with laser irradiation (980 nm, 1.0 W/cm^2^, 5 min). Subsequently, cells in both groups were incubated for another 0.5 h. The mitochondria were stained by MitoTracker™ Green at 200 nM for 30 min at 37 °C. Then cells in different groups were washed by cold PBS and were fixed by 4% paraformaldehyde for 30 min at 37 °C. Subsequently, cells were incubated with 0.2% Triton X-100 in PBS for 10 min to achieve good permeabilization. Immediately, the cells were blocked with blocking buffer for 2 h at room temperature. Primary antibody to the mitochondrial calcium uniporter (MCU) was incubated with the cells at 4 °C overnight. After the cells were washed three times by cold PBS, they were incubated with goat anti-rabbit IgG-Alexa 647 secondary antibody (1:200 dilution) for 2 h in room temperature. Red MCU was exited at 633 nm. Green mitochondrial was exited at 488 nm. The corresponding fluorescent images were obtained by CLSM. And the expression level of MCU was calculated by NIKON analyzer (version 5.20.00).

### In vitro evaluating the effect of NIR light-triggered acidification on ROS generation by CLSM and FCM

The HeLa cells were cultured as described above, treated with different treatment formulations (MU, MU-L, FMU-L, FMUP-L, and FMUP-L + Lip-1 with identical concentration of MU, 50 μg/mL) and then incubated overnight. As the replacement of nanoagents suspensions, identical volume of PBS was added into the cultural medium (DMEM) as the control group. The ROS were reacted with DCFH-DA for 20 min at 37 °C before NIR irradiation. Based on the Fenton reaction, Fe^2+^ could react with H_2_O_2_ to produce ROS more strongly and actively in acidic environment than that in weak alkaline environment after NIR irradiation (980 nm, 1.0 W/cm^2^, 5 min). Then the cellular ROS oxidized DCF can be used as indicator for ROS production. DCF was excited at 488 nm. The corresponding fluorescence images of cellular DCF at excitation wavelength of 510-555 nm were taken by CLSM. Moreover, the cells were collected and analyzed by FCM. The B525-FITC (gain value: 30) was chosen as the fluorescence channel for acquiring the fluorescence intensities of various groups (PBS, MU, MU-L, FMU-L, FMUP-L, and FMUP-L + Lip-1). Data were obtained from 20,000 cells for each sample.

### In vitro evaluating the effect of NIR light-triggered acidification on lipid peroxidation by CLSM and FCM

The HeLa cells were seeded and cultured in 35-mm glass-bottom dishes for overnight. Then the cells were incubated with different formulations (MU, MU-L, FMU-L, FMUP-L, and FMUP-L + Lip-1 with identical MU concentration, 50 μg/mL). As the replacement of nanoagents suspensions, identical volume of PBS was added into the cultural medium (DMEM) as the control group. After 12 h incubation, the lipid peroxidation and mitochondria in different groups were stained by lipid peroxidation probe (Liperfluo) and MitoTracker™ Red, respectively. In detail, the cells were incubated with lipid peroxidation probe (Liperfluo) at 10 μM for 30 min at 37 °C before NIR irradiation. Meanwhile, mitochondria were stained by MitoTracker™ Red at 200 nM for 30 min at 37 °C. After irradiation, the corresponding fluorescent images from various treatments could be obtained by CLSM. And the co-localization rates of lipid peroxidation (green) and mitochondria (red) in various treatments were calculated by NIKON analyzer. Moreover, the cells were collected and analyzed by FCM. The B525-FITC (gain value: 30) was chosen as the fluorescence channel for detecting the fluorescence intensity of various groups (PBS, MU, MU-L, FMU-L, FMUP-L, and FMUP-L + Lip-1). Data were obtained from 20,000 cells for each sample.

### In vitro evaluating the effect of NIR light-triggered acidification on ferroptosis by western blotting

In brief, HeLa cells were seeded in 48-well plates at a density of 1 × 10^6^ cells per well for attaching overnight. Then the cells were treated with different formulations (MU, MU-L, FMU-L, and FMUP-L with identical MU concentration, 50 μg/mL). As the replacement of nanoagents suspensions, identical volume of PBS was added into the cultural medium (DMEM) as the control group. The expressions of GPX4 proteins were evaluated by western blot for ferroptosis. Cytoplasm protein was extracted by Minute TM Cytoplasmic and Nuclear Extraction Kit (Invent Biotechnologies, USA). Protein concentration of each groups were measured by the BCA method. The protein was incubated with rabbit anti-human polyclonal antibody (1:2000). The protein antibody complexes were detected using the HRP (Horseradish peroxidase) conjugated secondary antibody (1:2000) (Solarbio, China). The images of western blotting were obtained using a Multicolor fluorescent gel imaging system with associated software (DNR MF ChemiBIS3.2, Israel, version 7.0.12).

### In vitro evaluating the effect of NIR-triggered acidification on mitochondrial membrane potential by FCM

In short, HeLa cells were seeded in 48-well plates at a density of 1 × 10^6^ cells per well for attaching overnight. Then the wells were treated with different formulations (MU, MU-L, FMU-L, and FMUP-L, equal concentration of MU 50 μg/mL) and then incubated for 12 h. As the replacement of nanoagents suspensions, identical volume of PBS was added into the cultural medium (DMEM) as the control group. After NIR irradiation (980 nm, 1.0 W/cm^2^, 5 min), the probe (JC-1) for measuring mitochondrial membrane potential (10 μg/mL) incubated with cells for 10 min at 37 °C. Next, the cells were washed by cold PBS with three times before collecting cells. And the mitochondrial membrane potential was determined on flow cytometer. The receiving emission wavelength of the JC-1 monomer is 529 nm and the receiving emission wavelength of the JC-1 aggregate is 590 nm. Data were obtained from 15000 cells for each sample.

### Evaluating the effect of NIR-triggered acidification on mitochondrial morphology by CLSM

The HeLa cells were seeded and cultured in 35-mm glass-bottom dishes for adhesion. Then the cells were treated with different formulations (MU, MU-L, FMU-L, and FMUP-L with identical MU concentration, 50 μg/mL). As the replacement of nanoagents suspensions, identical volume of PBS was added into the cultural medium (DMEM) as the control group. After 12 h incubation, cells in irradiation-involved groups were treated with NIR laser (980 nm, 1.0 W/cm^2^, 5 min). After that, cells in corresponding groups were incubated for another 0.5 h. After NIR irradiation, mitochondria in cells were stained by MitoTracker™ Green at 200 nM for 30 min at 37 °C. Then cells of different groups were washed by cold PBS and were fixed by 4% paraformaldehyde for 30 min at 37 °C. Next, Cells were incubated with 0.2% Triton X-100 in PBS for 10 min to achieve good permeabilization. Immediately, the cells were blocked with blocking buffer for 2 h at room temperature. Primary antibody to the mitochondrial membrane pore-associated protein Cyp D was incubated with the cells at 4 °C overnight. After the cells were washed three times by cold PBS, they were incubated with goat anti-rabbit IgG-Alexa 647 secondary antibody (1:200 dilution) for 2 h at room temperature. Green mitochondrial was excited at 488 nm. Red Cyp D was exited at 633 nm. The corresponding fluorescent images of mitochondrial and Cyp D were taken by CLSM. And the co-localization of these were calculated by NIKON analyzer.

### Evaluating the effect of NIR-triggered acidification on mitochondrial morphology by TEM

In short, HeLa cells were seeded in 48-well plates at a density of 1 × 10^6^ cells per well for attaching overnight. Then the wells were treated with different formulations (MU, MU-L, FMU-L, and FMUP-L, equivalent MU concentration, 50 μg/mL) and then incubated for 12 h. As the replacement of nanoagents suspensions, identical volume of PBS was added into the cultural medium (DMEM) as the control group. Cells in irradiation-involved groups were treated with NIR laser (980 nm, 1.0 W/cm^2^, 5 min). After that, cells in corresponding groups were incubated for another 0.5 h. Before TEM imaging, cells were fixed, dehydrated and stained as described in the above section with the detailed procedure reported previously^56^. Images of cells were obtained using TEM with associated software (JEOL JEM-1400, Japan, version 1.7.18.2349).

### Evaluating the effect of NIR-triggered acidification on mitochondrial function

For determining the function of mitochondria, the standard method was employed to measure the ATP production. In detail, HeLa cells were seeded in 96-well plate at a density of 1.5 × 10^4^ cells and cultured overnight for allowing attachment. Then the cells were incubated with different formulations (MU, MU-L, FMU-L, and FMUP-L) with equal concentration of MU (50 μg/mL) for another 12 h. As the replacement of nanoagents suspensions, identical volume of PBS was added into the cultural medium (DMEM) as the control group. Subsequently, cells were collected for detecting the ATP production. According to the kit instructions, the absorbance of each well at 340 nm was measured by automatic microplate reader with associated software (version 1.6.19.2) and the amount of ATP was calculated by:

1 $$ATP\,(\mu {{{{{\rm{mol}}}}}}/{10}^{5}\,{{{{{\rm{cells}}}}}})=\frac{{A}_{{{{{{\rm{sample}}}}}}}-{A}_{{{{{{\rm{control}}}}}}}}{{A}_{s\tan drad}-{A}_{{{{{{\rm{control}}}}}}}}\ast 0.125$$

where *A*_sample_ is the absorbance of test group, *A*_control_ is the absorbance of control group, and *A*_standrad_ is the absorbance of standard.

### CCK-8 cytotoxicity assay for different formulations

The cytotoxicity was determined using the CCK-8 (Beyotime, China) assay. Considering that the three cell lines (HeLa, 4T1 or HepG 2) highly express folate receptors^57–59^, we chose them for investigating the targeting ability of FMUP. Briefly, HeLa, 4T1 or HepG 2 were seeded in 96-well plate at a density of 1.5 × 10^4^ cells and cultured overnight for allowing cell attachment. Then the cells were treated with different formulations (MU, MU-L, FMU-L, and FMUP-L, respectively, with identical MU concentration ranging from 0 to 100 μg/mL in each case) and then incubated for another 12 h. Subsequently, NIR light irradiation (980 nm, 1.0 W/cm^2^, 5 min per well) was performed in those light-triggering-involved groups. To explore the effect of incubation time on cell viability, various incubation time duration (12 h, 24 h, and 48 h) were executed after laser irradiation. Moreover, in order to investigate the irradiation times on cell viability, the cells received the second irradiation after 24 h incubation. CCK-8 test solution was added to each well of the plate (The volume of the CCK-8 test solution in each well is one-tenth of the total volume) and incubated for another 3 h. The absorbance of each well at 450 nm was measured by automatic microplate reader with associated software (version 1.6.19.2). And the cell viability was calculated by:

2 $${{{{{\rm{Cell}}}}}}\,{{{{{\rm{viability}}}}}}\,( \% )=\frac{{A}_{{{{{{\rm{sample}}}}}}}-{A}_{{{{{{\rm{blank}}}}}}}}{{A}_{0}-{A}_{{{{{{\rm{blank}}}}}}}}\ast 100$$

where *A*_sample_ is the absorbance of wells with cells, CCK-8 solution and drug solution, *A*_blank_ is the absorbance of wells with medium and CCK-8 solution but no cells, and *A*_0_ is the absorbance of wells with cells, CCK-8 solution but no drug solution.

### Assessment of NIR-triggered acidification on apoptosis by CLSM

The HeLa cells were seeded and cultured in 35 mm glass-bottom dishes for adhesion. Then the cells were treated with different formulations (MU, MU-L, FMU-L, and FMUP-L, equivalent MU concentration, 50 μg/mL). As the replacement of nanoagents suspensions, identical volume of PBS was added into the cultural medium (DMEM) as the control group. After 12 h incubation, cells in both groups were treated with laser irradiation (980 nm, 1.0 W/cm^2^, 5 min). After that, cells in both groups were incubated for another 0.5 h. After NIR irradiation, nuclei were stained by Hoechst 33342 with 20 min at 37 °C. Then cells of different groups were washed by cold PBS and were fixed by 4% paraformaldehyde for 30 min at 37 °C. Subsequently, Cells were incubated with 0.2% Triton X-100 in PBS for 10 min to achieve good permeabilization. Immediately, the cells were blocked with blocking buffer for 2 h at room temperature. Primary antibody to the caspase-3 was incubated with the cells at 4 °C overnight. After the cells were washed three times by cold PBS, they were incubated with goat anti-rabbit IgG-Alexa 647 secondary antibody (1:200 dilution) for 2 h at room temperature. Blue nuclei were excited at 405 nm. Pink caspase-3 was exited at 633 nm. The corresponding fluorescent images were taken by CLSM. And the expression level of caspase-3 was calculated by NIKON analyzer.

### In vitro evaluation of cellular apoptosis by Live/dead assay

HeLa cells were cultured as described above, treated with different formulations (MU, MU-L, FMU-L, and FMUP-L, with identical MU concentration, 100 μg/mL) and then incubated for 12 h. As the replacement of nanoagents suspensions, identical volume of PBS was added into the cultural medium (DMEM) as the control group. The cells were stained by Calcein-AM/EthD-1 staining working solution for 20 min at 37 °C. Green (494/517 nm) viable cells can be stained by Calcein AM, while red (528/617 nm) dead cells can be stained by EthD-1.

### In vivo evaluation of tumor targeting

Balb/c-nu mice (4–6-week-old female) were bought from Vital River Laboratories (Beijing, China) and used for animal experiments directly. The tumors were obtained by injecting female mice with HeLa cells (8.0 × 10^6^ cells in 100 μL of PBS). In order to observe the biodistribution of different formulation in vivo, equivalent various MOFs (MUP/Cy7 or FMUP/Cy7) were intravenously injected to HeLa tumor-bearing mice and their biodistribution were observed by using animal imaging system with associated software (Kodak FX Pro, Japan, version 5.4.2.18893) at different time point. Then the tumors and organs were excised and imaged after 48 h. Due to MRI property of Fe^3+^, mice in different groups were imaged by MRI with associated software (BioSpec70/20USR, America, version 6.01) for tracking and diagnosis at tumor site before and after injection.

### Urine and feces sample collection and analysis

The model mice were randomly divided into two groups (*n* = 3). The mice were i.v. injected with FMUP (200 μL, 1 mg/mL). The urine and feces were collected at 48 h. Other three mice without injection were used as control. The mice were allowed to access food and water freely. Both urine and feces samples were digested by aqua regia and diluted by water for analysis of Y element, which could reflect the amount of our nanoagents.

### In vivo evaluation of anticancer efficacy in HeLa model

For investigating the antitumor effect of different treatment formulations, HeLa tumor-bearing mice were treated when the average tumor volume reached approximately 80 mm^3^. The mice were randomly divided into five groups (each group *n* = 6). Tumor volumes (V) were measured for five groups of mice that were intravenously administered with PBS, MU, MU-L (980 nm, 1.0 W/cm^2^, 5 min), FMU-L (980 nm, 1.0 W/cm^2^, 5 min), and FMUP-L (980 nm, 1.0 W/cm^2^, 5 min) until day 26. Note that mice in the groups with the involvement of light irradiation underwent treatment of irradiation (980 nm, 1.0 W/cm^2^, 5 min) twice, at 8 h and 32 h, respectively, after a single intravenous administration. Finally, the tumor sizes and survival percent were measured every 2 days and the experimental endpoint was defined as either death or the tumor size greater than 1500 mm^3^. Tumor volumes were calculated by:

3 $$V=\frac{L\times {W}^{2}}{2}$$

where *L* is the longest and *W* is the shortest tumor diameter (mm).

### Antitumor study in PDX model

The PDX tumor samples were s.c. transplanted into the axilla of the NTG mice to establish the PDX model. In detail, the sample of PDX was produced from one patient. The tumor tissue was divided into five pieces and inoculated into the five NTG mice. Until the tumor grew to 1000 mm^3^, the tumors from tumor-bearing NTG mice were collected and inoculated into 10 mice as the second passages. After that, we repeated the above steps to inoculate NTG mice for subsequent experiments. Six mice (two groups, 3 mice per group) were used to evaluate the FA-mediated targeting capacity. Six mice (two groups, 3 mice per group) were used to evaluate the acidification ability of nanoagents. Twelve mice (two groups, 6 mice per group) were used to evaluate the antitumor ability of nanoagents. Two weeks later, when the tumors were visible (~150 mm^3^), the mice of these groups were i.v. injected with PBS and FMUP, respectively. Eight hour later, the mice in FMUP + L group were anaesthetized and the tumors of them were illuminated with a 980-nm laser (1.0 W/cm^2^, 5 min). Note that mice in the groups with the involvement of light irradiation underwent treatment of irradiation (980 nm, 1.0 W/cm^2^, 5 min) twice, at 8 h and 32 h, respectively, after a single intravenous administration. Finally, the tumor sizes and survival percent were measured every two days, and the experimental endpoint was defined as either death or the tumor size greater than 1500 mm^3^. The mice bearing tumor over 1500 mm^3^ received euthanasia, rather than being left to die due to excessive tumor burden. The biodistribution study was administered in the same ways as we used in the corresponding studies. And the acidification study was investigated by the multiphoton laser confocal microscopy with associated software (Leica, Germany, version 3.0). The evaluation of ROS generation in tumor was measured by FCM. Besides, the investigation for calcium influx in tumor was detected by frozen section staining using Fluo-3. Moreover, the evaluation of mitochondria damage was measured via immunohistochemical staining. The haematoxylin and eosin (H&E) staining of tumor sections for the study was administered in the same way. The ethics with informed consent was obtained from Shanghai Tongren Hospital medical ethics committee (2019-032-01 and 2021-003-01) in accordance with the 1964 Declaration of Helsinki and 1982 International Ethical Guidelines for Human Biomedical Research.

### Evaluation of acidification on tumor by animal imaging system

Before measuring the pH values in tumor sites, the standard solutions with different pH values (5.0, 5.5, 6.0, 6.5, 7.0, and 7.5) were mixed with SNARF^®^-1 at a volume ratio of 1:100, respectively. And the fluorescence at 580 nm and 640 nm of various standard solutions was determined by animal imaging system with associated software (Kodak FX Pro, Japan, version 5.4.2.18893). As a result, the calibration curve was obtained. To estimate the pH in HeLa the tumor site, mice with different treatments (PBS, MU, MU-L, FMU-L, and FMUP-L) were intravenously injected with a pH-sensitive fluorescent dye (SNARF®-1) at 20 min before imaging. After that, mice in the light-triggering groups received NIR light irradiation. Note that pH in tumor was evaluated by the animal imaging system after laser irradiation rather than on the second day due to rapid metabolism of small molecule probe (SNARF®-1) in the body. Finally, the pH value in tumor sites was determined by the ratio of the fluorescence at 580 nm and 640 nm.

### Evaluation of acidification on ROS production in tumor

In order to investigate the generation of ROS in situ, DCFH-DA was intratumorally administered before imaging by multiphoton laser confocal scanning microscopy. After that, the mice were anesthetized and then the mice’s tumor sites were irradiated by NIR laser (980 nm, 1.0 W/cm^2^, 5 min). Subsequently, the green ROS was detected and measured by multiphoton laser confocal scanning microscopy with associated software (Leica, Germany, version 3.0) and further quantitative analysis for different treatments.

### Evaluation of the synergistic effect on mitochondria by immunohistochemical section

After the completion of nanoagent administration, three mice were taken from each group for immunofluorescence section and then imaging. Subsequently, the corresponding quantitative analysis was acquired based on the representative images. The expression of uncoupling protein 2 (UCP 2) in mitochondrial membrane induced by the synergistic effect was also verified by immunohistochemistry. The images of immunohistochemistry (IHC) were visualized by using an automatic multispectral imaging system with associated software (Vectra II, PerkinElmer, version 2.0.7.1).

### Evaluation of the synergistic effect on tumor inhibition by immunohistochemical section

After the completion of nanoagent administration, three mice were taken from each group for immunofluorescence section and then imaging. Subsequently, the corresponding quantitative analysis was acquired based on the representative images. Proliferating cell nuclear antigen-K_i_67 was used for determining nucleus proliferating of tumor tissue.

### Safety evaluation of different treatments

For further investigate the safety of different formulations in vivo, the body weight of mice in each groups were recorded until 60 days. Besides, the serum levels of urea nitrogen (BUN), lactate dehydrogenase (LDH), alanine aminotransferase (ALT), aspartate transaminase (AST), and alkaline phosphatase (ALP) were analyzed by using an automated analyzer (Hitachi Ltd Hitachi-917, Japan). The main organs (heart, liver, spleen, lung, and kidney) were sliced and stained by hematoxylin-eosin (H&E) staining.

### Animal care

Balb/c-nu mice (4–6-week-old female) were obtained from Vital River Laboratories (Beijing, China) and 6-week old (females) NOD.Cg-Prkdc^scid^Il2rg^tm1Sug^/ShiJic (NTG) mice were purchased from SiPeiFu Biotechnology Co., Ltd (Beijing, China). This study was performed in strict accordance with the Regulations for the Care and Use of Laboratory Animals and Guideline for Ethical Review of Animal (China, GB/T 35892-2018). All animal experiments were reviewed and approved by the Animal Ethics Committee of the Institute of Process Engineering (approval ID: IPEAECA2019318). All the model mice were raised in a standard environmentally controlled room (23 °C, with 55 ± 5% humidity and under a 12 h–12 h light–dark cycle).

### Statistical analysis

All the data are presented as the mean ± SD. Statistical analysis was performed with Prism 8.0 software (GraphPad Software) by an unpaired Student’s *t* test, Log-rank test, one-way ANOVA.

### Reporting summary

Further information on research design is available in the Nature Research Reporting Summary linked to this article.

## Supplementary information

Supplementary Information

Reporting Summary

## Data Availability

The main data supporting the results in this study are available within the paper and its Supplementary Information. Source data for the figures in the main text are available at Figshare (10.6084/m9.figshare.16586456). Source data for the figures in the Supplementary Information are available at Figshare (https://figshare.com/articles/dataset/MOFs-Based_Nanoagent_Enables_Dual_Mitochondrial_Damage_in_Synergistic_Antitumor_via_Oxidative_Stress_and_Calcium_Overload/16586456).
