# Supplementary material for: MOFs-based nanoagent enables dual mitochondrial damage in synergistic antitumor therapy via oxidative stress and calcium overload
Source: Nat Commun. 2021 Nov 4;12:6399. doi: 10.1038/s41467-021-26655-4 (PMC8569165; doi:10.1038/s41467-021-26655-4)
Supplement: Supplementary file 1 — Supplementary Information [file 41467_2021_26655_MOESM1_ESM.pdf]

Supplementary Information

MOFs-Based Nanoagent Enables Dual Mitochondrial Damage in Synergistic Antitumor Therapy via Oxidative Stress and Calcium Overload

Authors: Weier Bao[1,2], Ming Liu[1], Jiaqi Meng[1,2], Siyuan Liu[1], Shuang Wang[2], Rongrong Jia[3], Yugang Wang[3], Guanghui Ma[2,4]* Wei Wei[2,4]*, Zhiyuan Tian[1]*

Affiliations:

[1]School of Chemical Sciences, University of Chinese Academy of Sciences, Beijing 100049, P. R. China.

[2]State Key Laboratory of Biochemical Engineering, Institute of Process Engineering, Chinese Academy of Sciences, Beijing 100190, P. R. China.

[3]Department of Gastroenterology, Shanghai Tongren Hospital, Shanghai Jiao Tong University School of Medicine, Shanghai 200336, P. R. China.

[4]School of Chemical Engineering, University of Chinese Academy of Sciences, Beijing 100049, P. R. China.

Correspondence and requests for materials should be addressed to Zhiyuan Tian (zytian@ucas.ac.cn) or Wei Wei (weiwei@ipe.ac.cn) or Guanghui Ma (ghma@ipe.ac.cn).

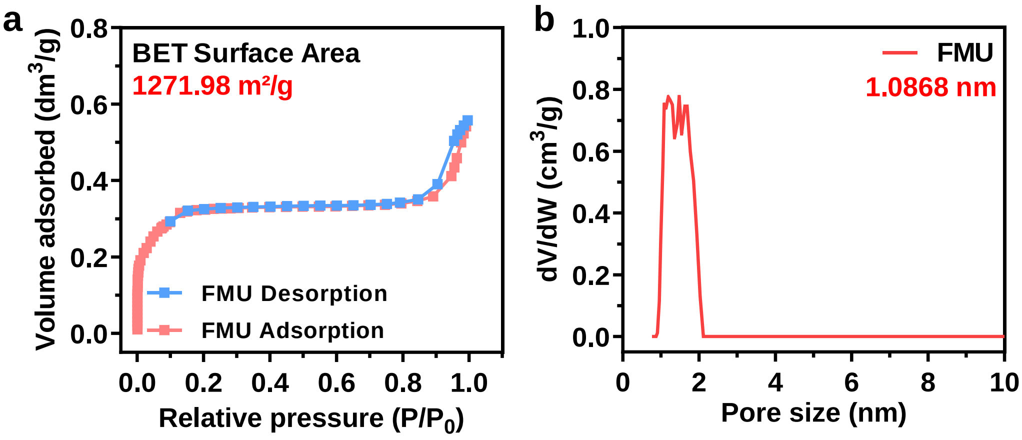

Supplementary Fig. 1 Characterizations of FMU nanoagents.

(a) Nitrogen adsorption-desorption isotherms for FMU.

(b) The Pore size distribution of FMU determined by the Barrett-Joyner-Halenda method.

The large surface area and suitable pore size were favourable for high loading efficiency.

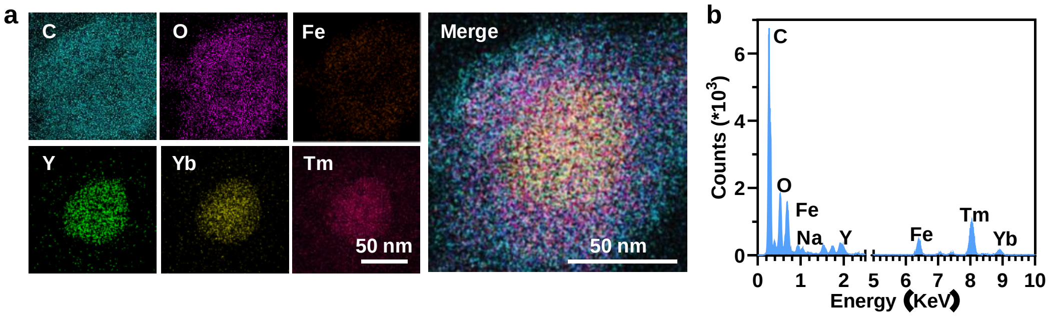

Supplementary Fig. 2 Element analysis of FMUP nanoagents.

(a) Element mapping images of FMUP (C, O, Fe, Y, Yb, and Tm). A representative image of three biologically independent samples from each group is shown in Figure.

(b) EDX element analysis of FMUP.

The core-shell structure was verified by the X-ray spectrometric analysis results. Specifically, the main elements (Y, Yb, and Tm) of UCNPs were found evenly distributed in the middle of the MOFs shell.

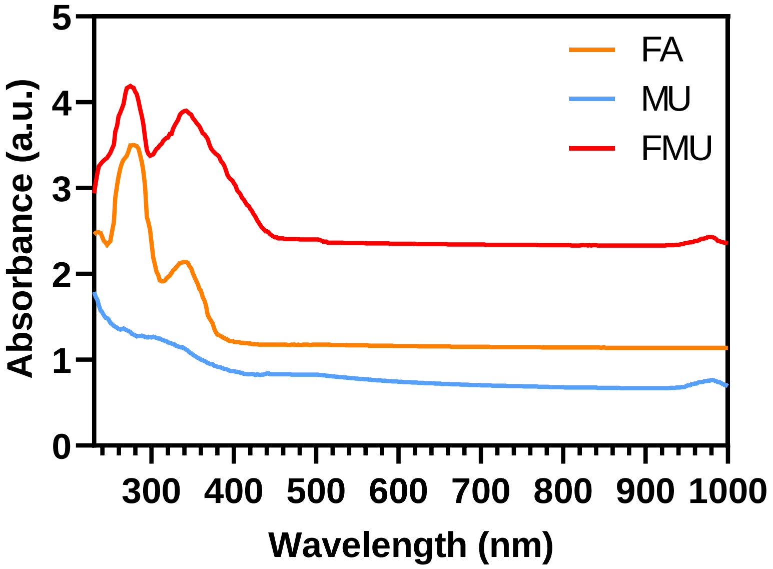

Supplementary Fig. 3 UV/vis absorption spectra of MOFs before and after FA decoration. The beaconing absorbance peak (at ~280 nm) indicated that FA moiety was successfully incorporated in the MOFs.

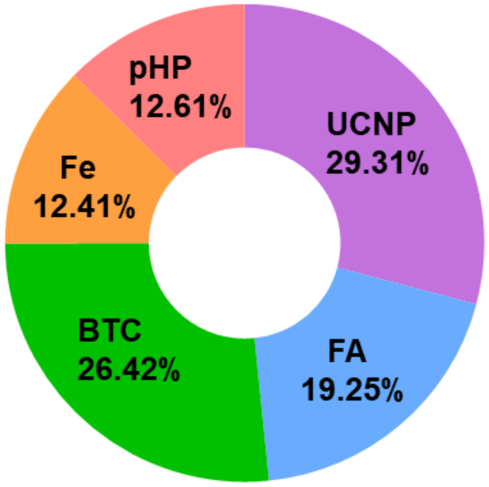

Supplementary Fig. 4 The weight percentages of constituent components in FMUP nanoagents.

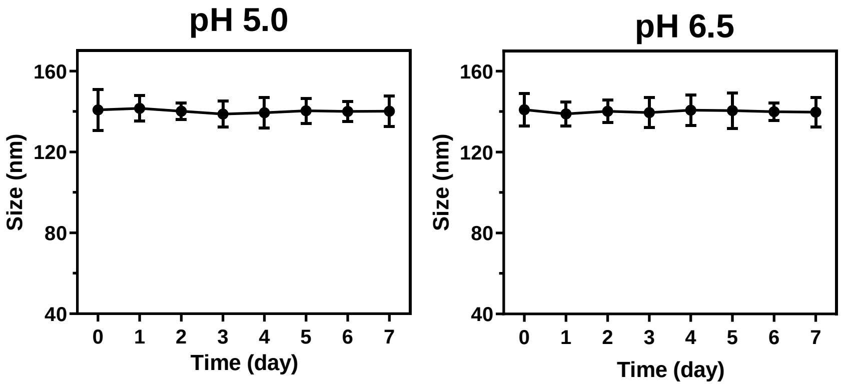

Supplementary Fig. 5 The stability of FMUP nanoagents in cell culture media with various pH (5.0 and 6.5).

FMUP exhibited excellent stability at various pH (5.0 and 6.5), indicating that the structure of FMUP was not affected by pH. Data represent mean values ± SD, n = 3 biologically independent samples in both figures.

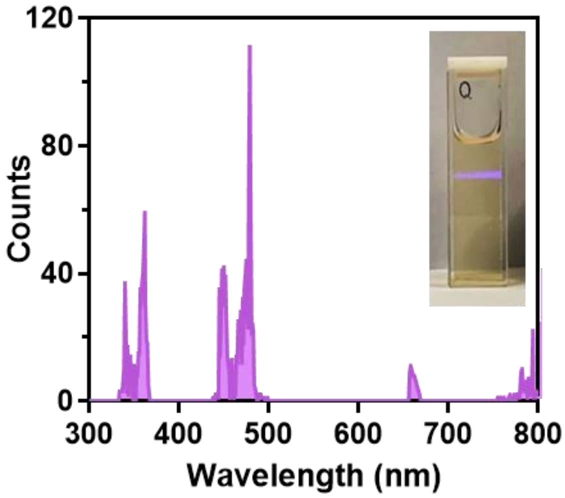

Supplementary Fig. 6 Full-wavelength luminescence emission spectrum and image of FMUP-dispersed aqueous sample upon NIR laser irradiation (980 nm).

The FMUP displayed characteristic UV (365 nm) emission feature upon irradiation of 980-nm laser, which was favorable for photochemically reducing the Fe^{3+} species and releasing H^+ from the pHP.

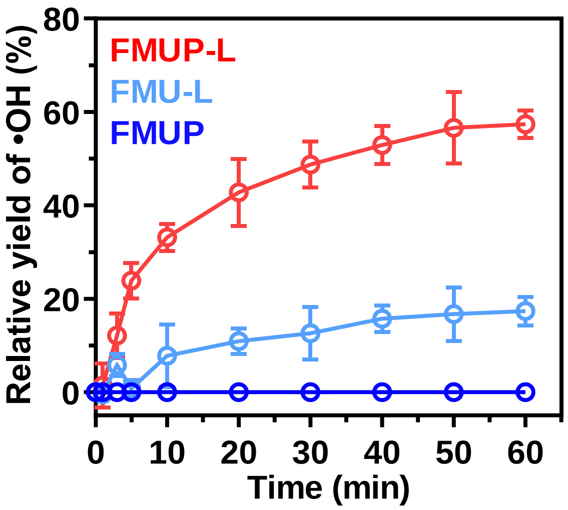

Supplementary Fig. 7 Relative yield of •OH *in vitro* in different groups.

The group of FMUP-L displayed much higher •OH generation efficiency as compared to other groups. Data represent mean values ± SD, n = 3 biologically independent samples in figure.

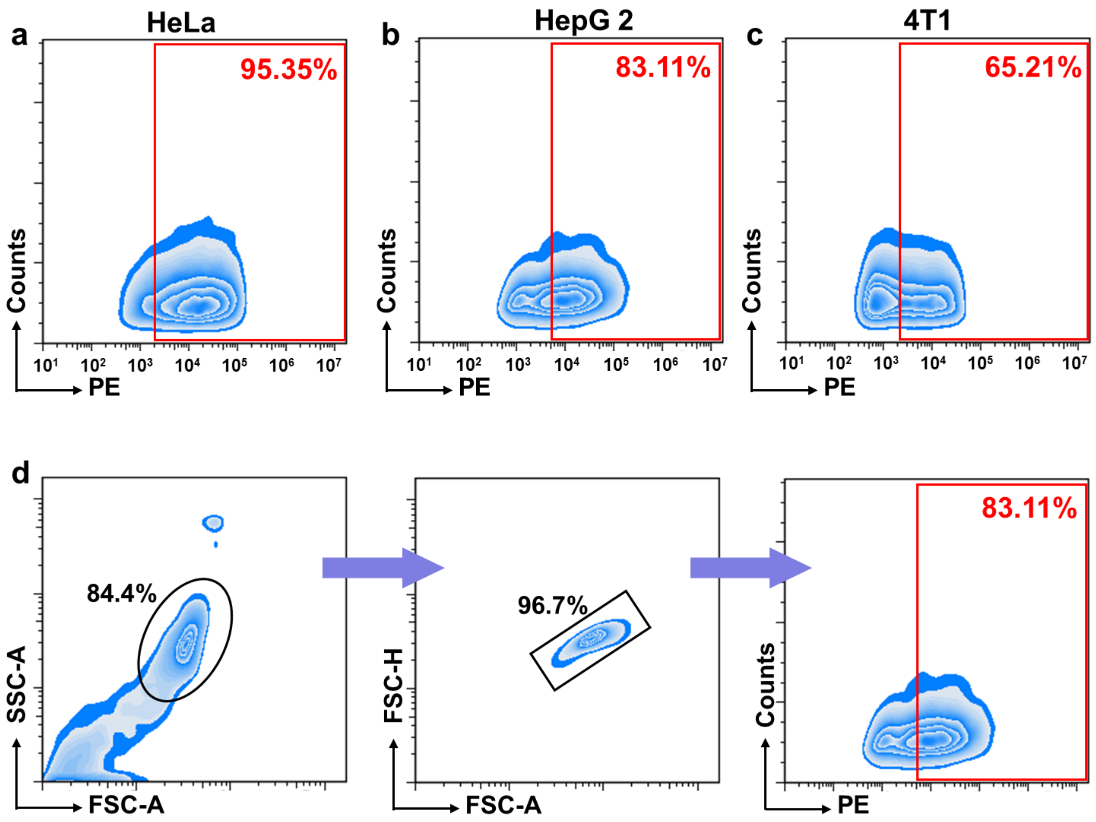

Supplementary Fig. 8 Evaluation of folate receptors (FR) levels on various cell lines by flow cytometry.

(a) The analysis of FR expression on HeLa cells using flow cytometry.

(b) The analysis of FR expression on HepG2 cells using flow cytometry.

(c) The analysis of FR expression on 4T1 cells using flow cytometry.

(d) Flow cytometry gating strategy for evaluating of FR levels on cells.

All three types of cells were found characterized with appreciably upregulated FR, which was expected to facilitate the cellular uptake of FA-functionalized FMUP nanoagents.

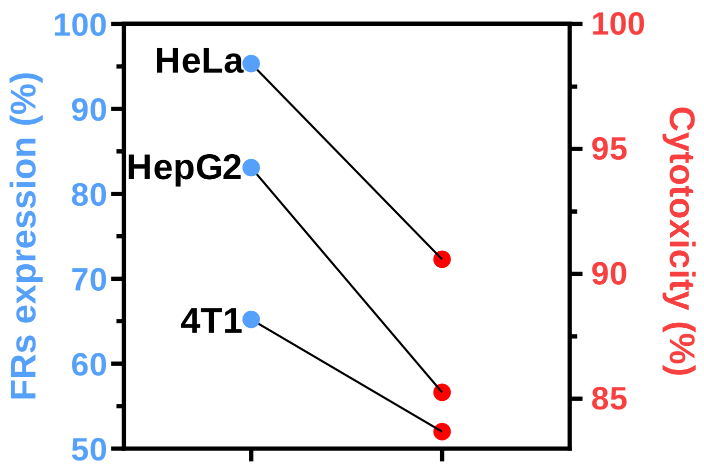

Supplementary Fig. 9 The analysis of the relationship of FR expression level vs. FMUP-L cytotoxicity level. Taking the dose of 100 μg/mL as an example, the cytotoxicity level definitely increased with upregulated expression of FR, indicating a clear correlation between them.

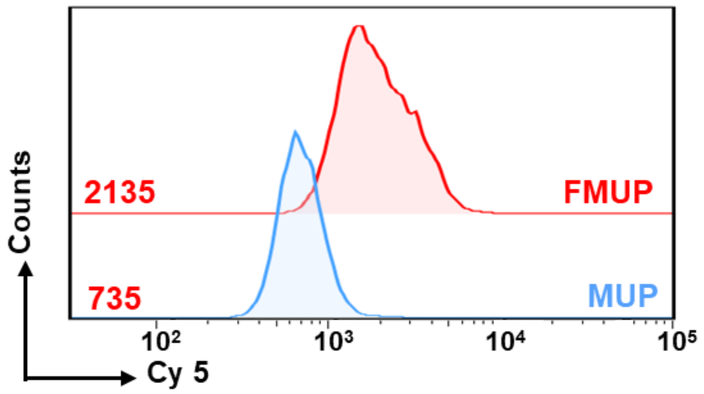

Supplementary Fig. 10 The flow cytometry analysis of FMUP and MUP nanoagents in HeLa cells.

The FMUP was more easily internalized by HeLa cells as compared to MUP, indicating the FA-enabled targeting ability of the nanoagents.

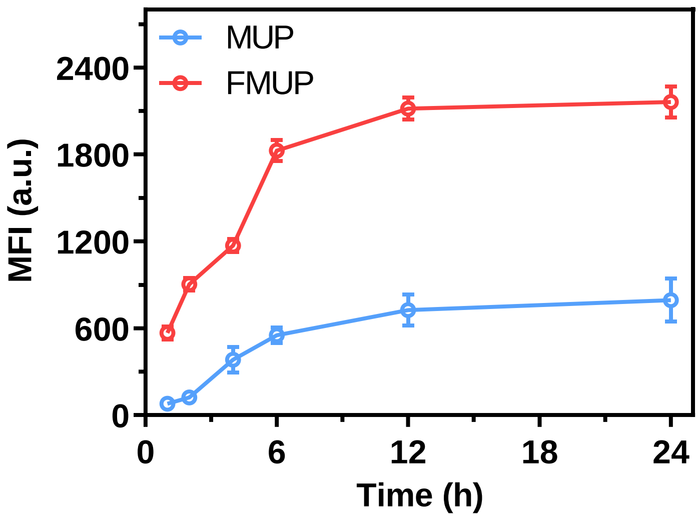

Supplementary Fig. 11 The analysis of internalization kinetics of different types of nanoagents in HeLa cells.

The amount of nanoagents internalized by HeLa cells reached saturation after 12 h. Data represent mean values ± SD, n = 3 biologically independent samples in figure.

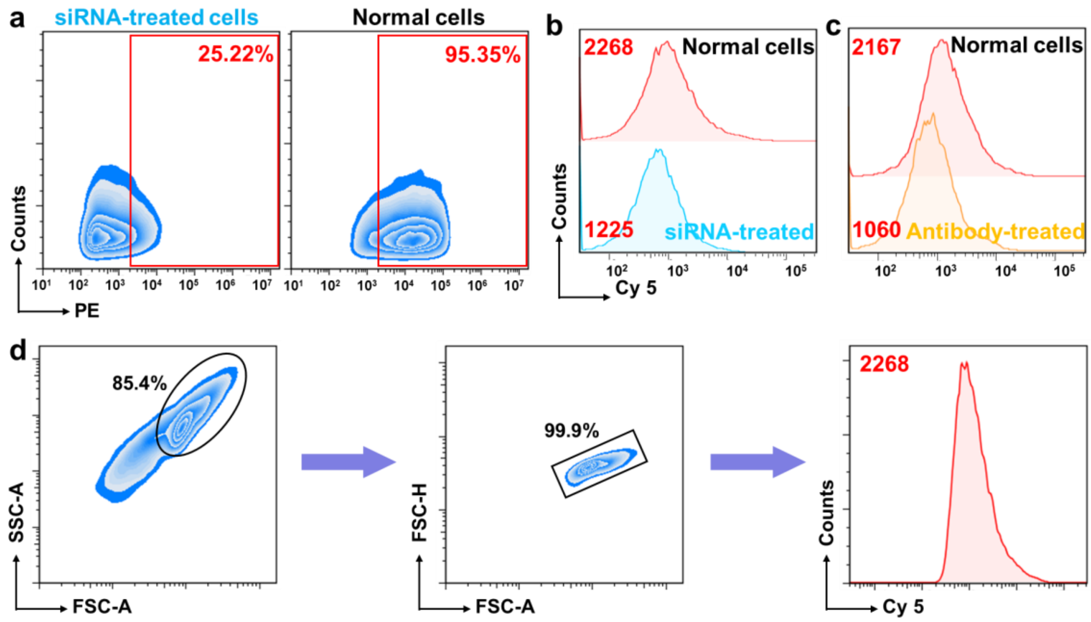

Supplementary Fig. 12 Evaluation of FR for FMUP internalization.

(a) The analysis of FR expression on normal HeLa cells and siRNA-treated HeLa cells by using flow cytometry.

(b) The flow cytometry analysis of FMUP internalization in siRNA-treated HeLa cells and normal cells.

(c) The flow cytometry analysis of FMUP internalization in antibody-treated HeLa cells and normal cells.

(d) Flow cytometry gating strategy for evaluating FMUP internalization in HeLa cells.

The beaconing fluorescence signals of FMUP dramatically decreased in siRNA-treated HeLa cells or antibody-treated HeLa cells, indicating the important role of FR for FMUP uptake.

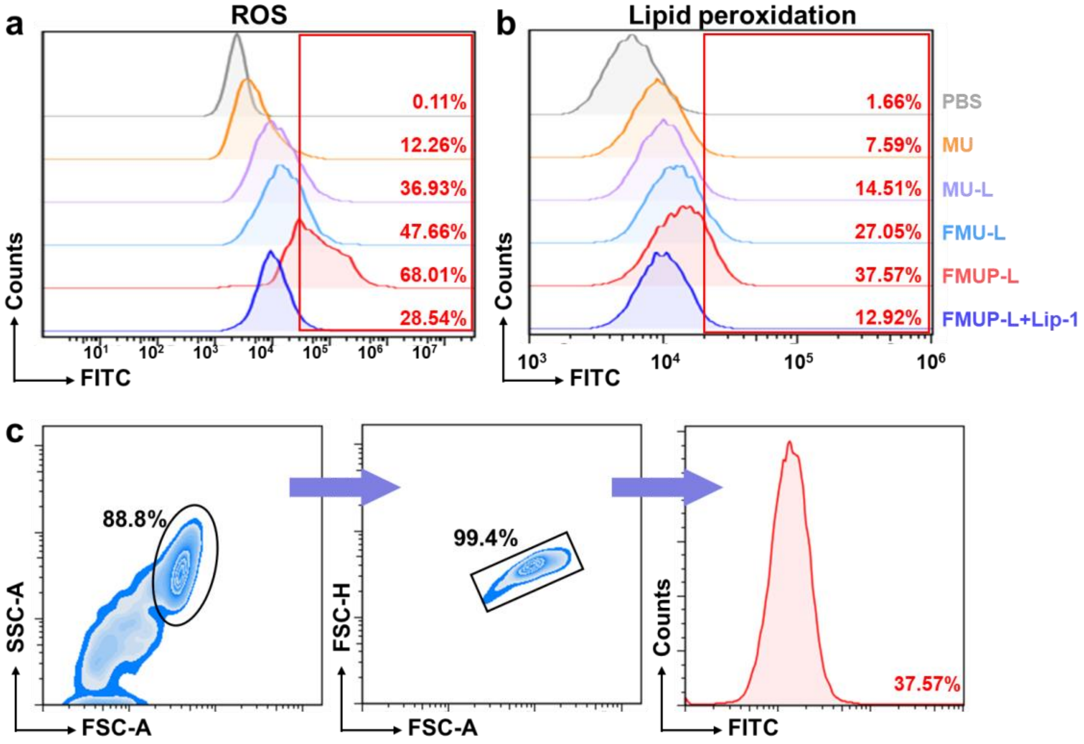

Supplementary Fig. 13 Evaluation of ROS production and lipid peroxidation in HeLa cells upon various treatments by flow cytometry.

(a) Quantitative analysis of ROS production by flow cytometry.

(b) Quantitative analysis of lipid peroxidation by flow cytometry.

(c) Flow cytometry gating strategy for evaluating ROS production or lipid peroxidation in HeLa cells.

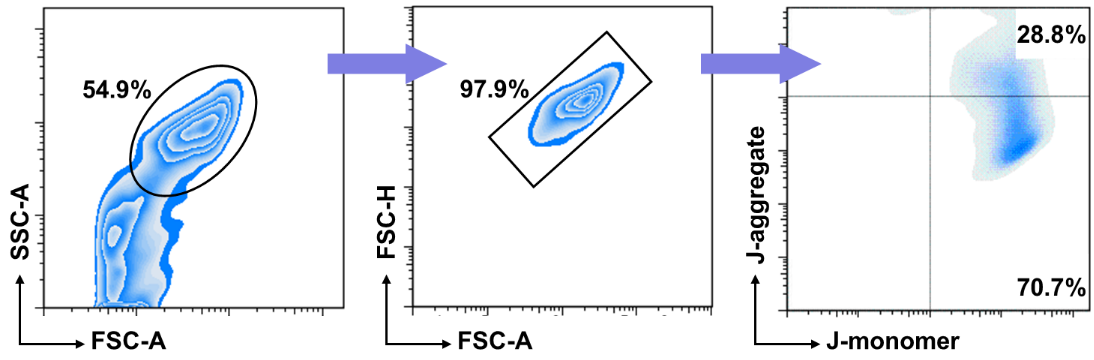

Supplementary Fig. 14 Flow cytometry gating strategy for Fig. 4b.

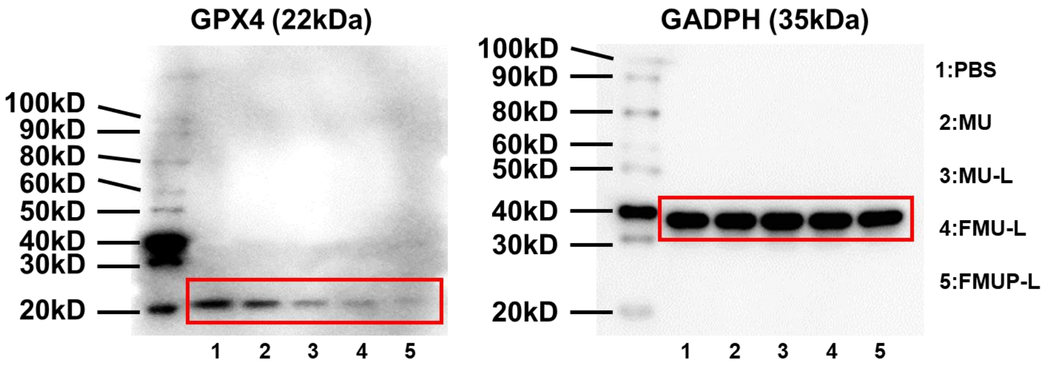

Supplementary Fig. 15 Whole western blotting of glutathione peroxidase (GPX4) and GADPH. The samples derive from the same experiment and that gels/blots were processed in parallel. A representative image of three biologically independent samples from each group is shown in Figure.

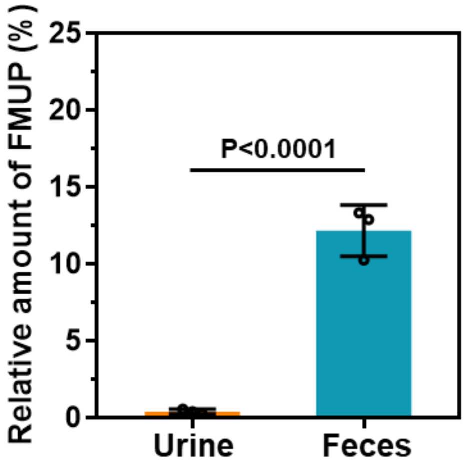

Supplementary Fig. 16 Quantification analysis of FMUP nanoagents in the urine and feces collected from model mice 48 h after injecting FMUP.

Most of nanoagents were excreted through feces rather than urine. Data represent mean values ±SD, n = 3 biologically independent samples in figure. Statistical significance was determined using two-tailed unpaired Student's t-test.

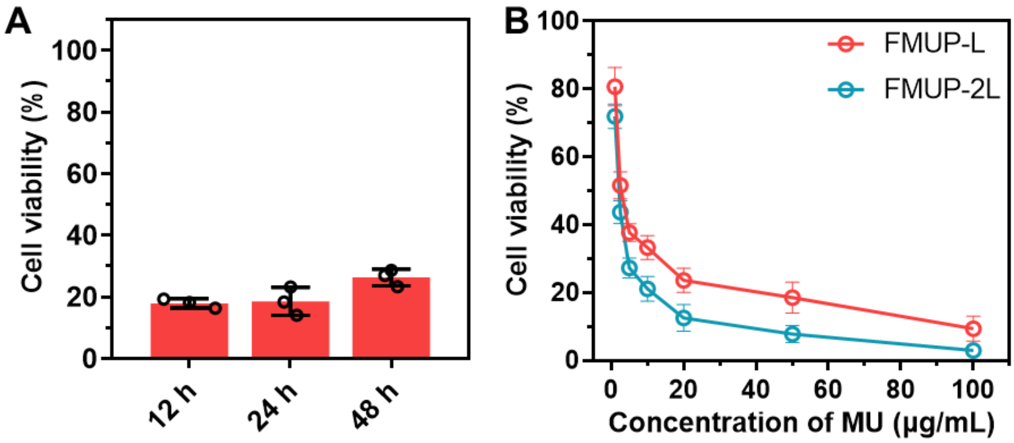

Supplementary Fig. 17 Evaluation of cytotoxicity of different treatment formulations.

(A) CCK-8 cytotoxicity analysis of HeLa cells treated with different incubation time.

(B) CCK-8 cytotoxicity analysis of HeLa cells treated with different irradiation times.

The cell viability was found appreciably affected by irradiation times. Data represent mean values ± SD, n = 3 biologically independent samples in both figures.

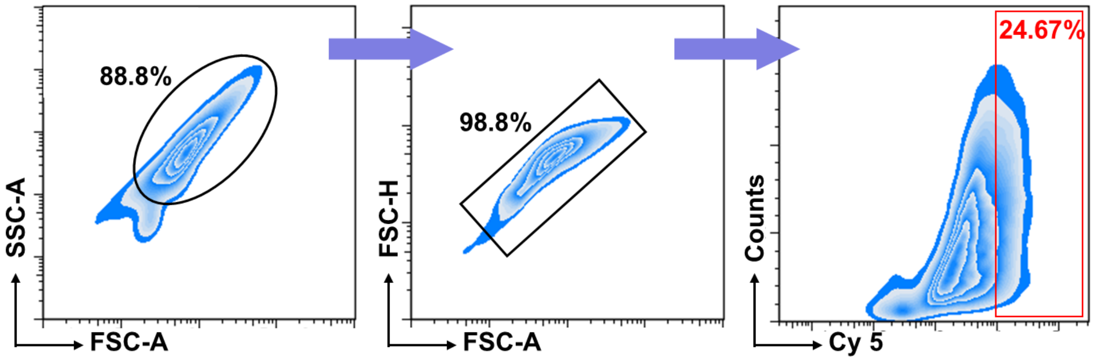

Supplementary Fig. 18 Flow cytometry gating strategy for Fig. 7e.

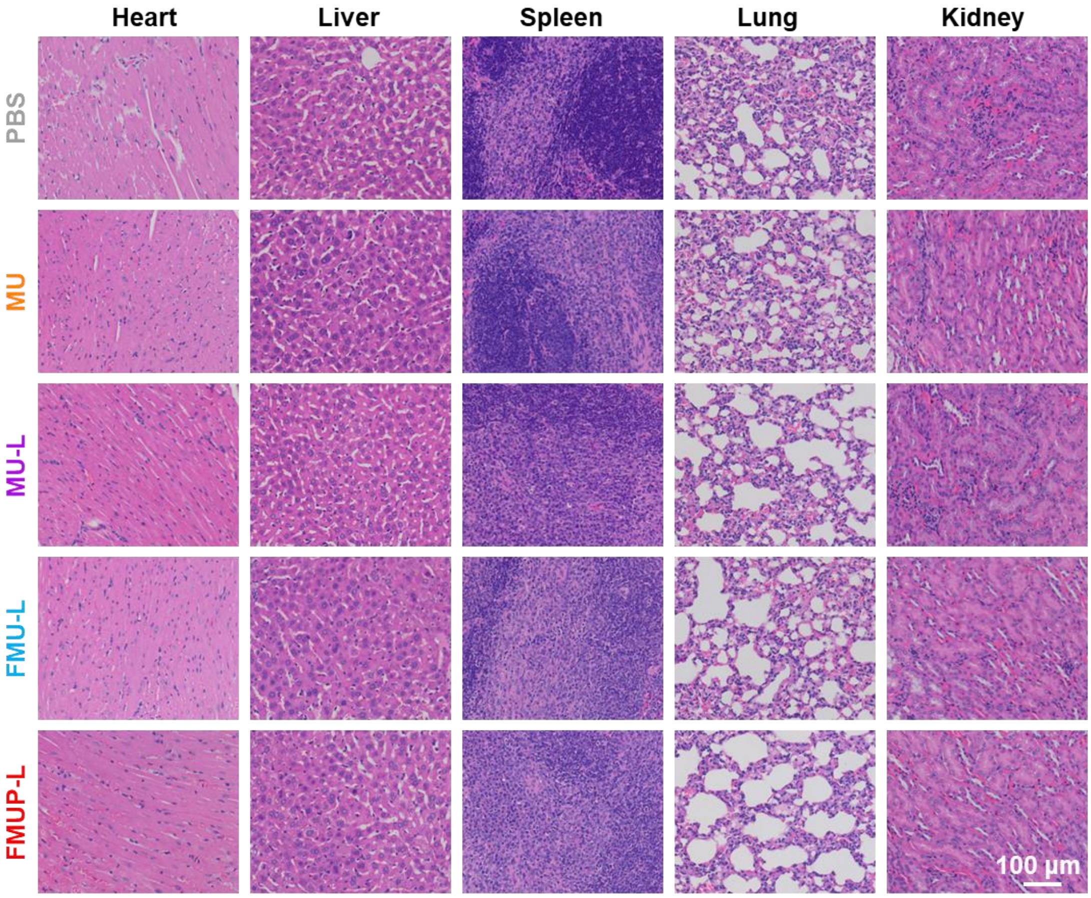

Supplementary Fig. 19 H&E-stained slice images of major organs from model mice upon different treatments. A representative image of three biologically independent samples from each group is shown in Figure.

No significant injury was observed in mice of all treatment groups, indicating the biosafety of the treatment formulations based on the NIR light-responsive nanoagents developed in this work.

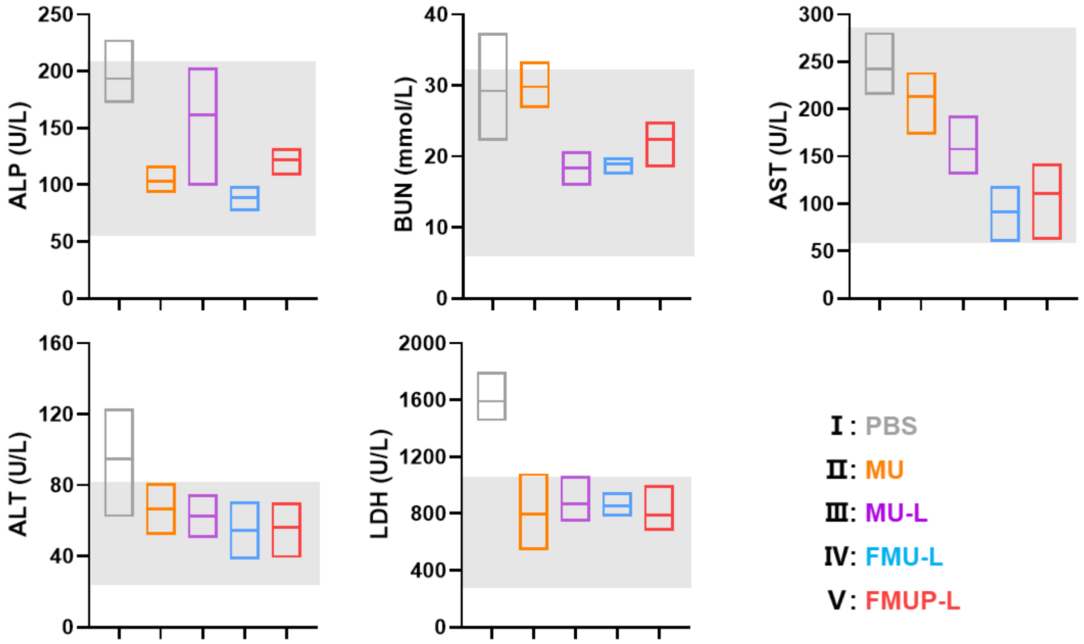

Supplementary Fig. 20 Blood biochemical analysis of treated mice in HeLa model.

Few abnormalities were found in all markers in model mice upon treatments, indicating that the good safety of our nanoagents. Gray areas represent the normal range of different biosafety indicators. All box plots represent minimum to maximum values and the average values in each group are exhibited by Mean values. Data represent mean values ± SD, n = 3 biologically independent samples in Figure.

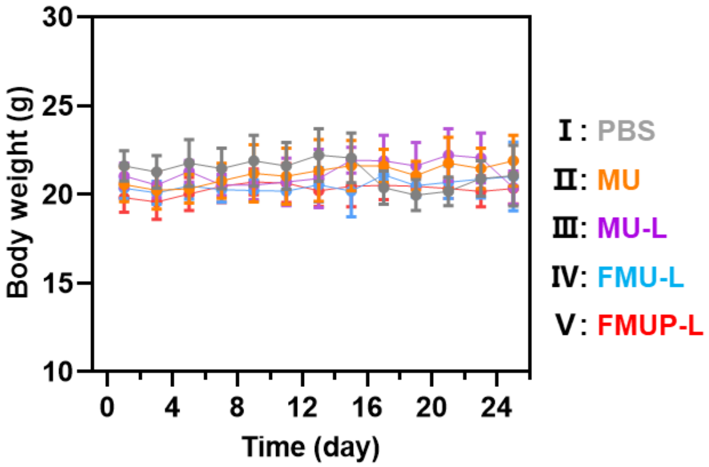

Supplementary Fig. 21 Body weight changes after different treatments.

The treatments with different formulations did not exert appreciable influence on the body weights of model mice, further confirming the safety of our MOFs-based nanoagents for anticancer therapy. Data represent mean values ± SD, n = 6 biologically independent samples in Figure.

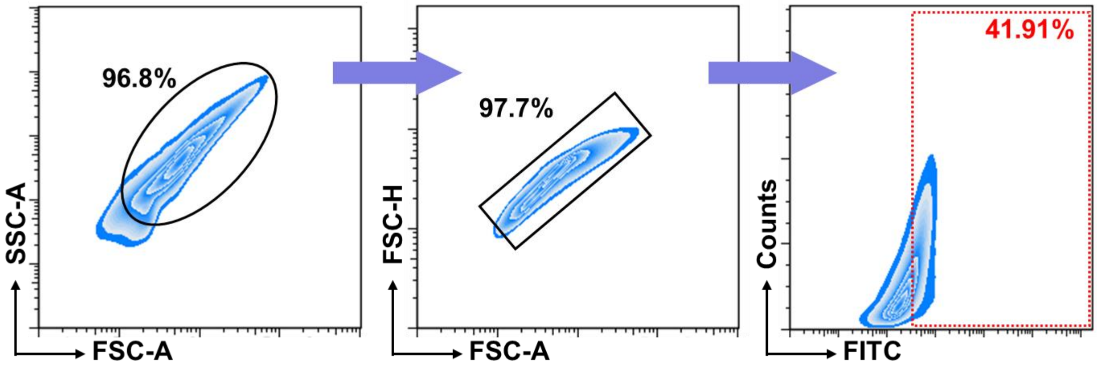

Supplementary Fig. 22 Flow cytometry gating strategy for Fig. 8g.
